# Development and External Validation of a Machine Learning Model for Risk Stratification of Presenteeism in Clinical Nurses: A Multicenter Cross‐Sectional Study

**DOI:** 10.1155/jonm/6257117

**Published:** 2026-09-09

**Authors:** Yumeng Zhang, Shaoting Yang, Qingfen Zeng, Honghong Wen, Heting Liang, Yunting Li, Shiming Huang, Huiming Gao, Chaoping Wang, Xiaoli Yuan

**Affiliations:** ^1^ Department of Nursing, Affiliated Hospital of Zunyi Medical University, Zunyi 563000, Guizhou, China, zmchospital.com.cn; ^2^ Faculty of Nursing, Zunyi Medical University, Zunyi 563000, Guizhou, China, zmc.edu.cn; ^3^ Department of Emergency, Tongren People’s Hospital, Tongren 554300, Guizhou, China

**Keywords:** clinical nurses, machine learning, network analysis, presenteeism, risk estimation model, risk stratification

## Abstract

**Background:**

Presenteeism impairs clinical nurses′ work performance and patient safety, yet externally validated risk estimation tools capturing nonlinear effects are lacking.

**Objectives:**

To develop and externally validate a machine learning‐based risk estimation model for presenteeism among clinical nurses, and to explore predictor roles across risk levels.

**Methods:**

This multicenter cross‐sectional survey recruited 19,729 clinical nurses from 153 hospitals across 9 cities in Guizhou, China (July–November 2025), using the Stanford Presenteeism Scale as the outcome. Predictors were selected via Least Absolute Shrinkage and Selection Operator regression and multiple linear regression. Nine machine learning models were developed (derivation: *n* = 13,323) and externally validated (*n* = 6406). Nurses were stratified into low‐, medium‐, and high‐risk groups by tertiles of predicted scores. The optimal model was interpreted using SHapley Additive exPlanations (SHAP) and partial dependence plots. Regularized partial correlation networks were estimated within each risk group.

**Results:**

Five predictors were retained: burnout (Maslach Burnout Inventory–General Survey, MBI‐GS), perceived social support (PSSS), organizational climate, workplace violence, and night shift involvement. The generalized additive model (GAM) performed optimally. SHAP showed burnout had the highest main effect, most pronounced in low‐ and high‐risk strata. Partial dependence plots revealed a linear increasing effect of MBI‐GS, a gently descending trend of PSSS, and an inverted U‐shaped relationship for organizational climate. Network analysis showed denser networks with more negative edges as risk escalated; department leadership/communication consistently served as a core node, while burnout node centrality increased markedly in the high‐risk group.

**Conclusions:**

This study developed and cross‐regionally validated a GAM‐based risk estimation model for clinical nurse presenteeism. The advantage of GAM over linear regression was its ability to capture nonlinear dose–response relationships, notably the inverted U‐shaped organizational climate association. Burnout was the strongest predictor across all risk levels. These findings provide preliminary evidence and visualization approaches that may inform risk‐stratified management of presenteeism, pending prospective validation.

**Implications for Nursing Management:**

A stepped, risk‐stratified approach to presenteeism may be considered for future evaluation: preventive resource building for low‐risk nurses, threshold monitoring for medium‐risk nurses, and enhanced organizational support for high‐risk nurses. Strengthening department leadership and communication warrants particular attention in intervention design.

## 1. Introduction

Presenteeism—the behavior of continuing to work despite health problems, often with reduced productivity—is prevalent among clinical nurses due to high workload, shift schedules, and low replaceability [1–4]. A meta‐analysis of 28 studies across 14 countries reported a global prevalence of approximately 49.2% among nurses [5]. In China, Shan et al. [6] estimated that nurse presenteeism causes an annual economic loss of approximately 4.38 billion RMB. Presenteeism not only erodes nurses′ physical and mental health [7–9] but also reduces care quality, increases patient safety risks [10, 11], and elevates turnover intention [12]. Its hidden health productivity loss exceeds that of explicit absenteeism [2, 13]. Accurately identifying core influencing factors and constructing scientifically sound risk estimation tools are, therefore, urgent priorities in nursing human resource management.

Previous studies have examined nurse presenteeism from multiple dimensions: burnout [7, 9], work stress [7, 14], and psychological capital [15, 16] at the individual level; fatigue [8] and night shift work [10] at the occupational level; and perceived social support [17, 18], perceived organizational support [19], and transformational leadership [20] at the organizational level. However, three limitations persist. First, most studies rely on linear regression or structural equation modeling, which cannot capture nonlinear dose‐response relationships or complex interaction effects [8, 9, 17]. Machine learning methods can detect such patterns directly from data and have shown superior performance in predicting nurse turnover [21, 22] and burnout [23]. For example, Žiedelis et al. [21] used an artificial neural network to predict turnover intention, revealing nonlinear age effects and predictor interactions via SHapley Additive exPlanations (SHAP) analysis. Second, although Wu et al. [20] constructed a nomogram for ICU nurse presenteeism, it was based on logistic regression and lacked independent external validation. For the broader clinical nurse population, externally validated machine learning‐based tools capturing nonlinear relationships are still absent. Third, most studies estimate only average effects without differentiating how factor contributions structurally differ across risk levels [18, 24].

Moreover, existing models rarely combine in‐depth interpretability with visualization. SHAP can address the “black box” problem by computing each feature’s marginal contribution [21, 25], but has not been applied to heterogeneous risk‐level contributions in presenteeism. Partial dependence plots (PDP) can visualize nonlinear dose‐response patterns and interactions [26], yet their combined use with SHAP remains rare in nursing research. Network analysis can reveal complex topological structures by identifying core nodes and bridge variables [27–30], but has not been applied to presenteeism risk‐stratified association structures [29, 31].

Therefore, this study aimed to construct and externally validate a machine learning‐based risk estimation model for clinical nurse presenteeism. By integrating SHAP, PDP, and network analysis, we sought to reveal nonlinear dose‐response relationships, synergistic interaction patterns, and risk‐dependent reorganization of variable associations, providing an evidence base to inform the development of risk‐stratified intervention strategies, pending prospective validation.

## 2. Methods

### 2.1. Study Setting and Participants

A multicenter cross‐sectional survey recruited clinical nurses from 153 hospitals across 9 cities in Guizhou Province, China (July 1–November 15, 2025). Nursing managers received standardized online training on the study protocol and quality control. Electronic questionnaires with mandatory response checks, logic skip functions, and IP address restrictions were distributed via an online platform after informed consent.

Inclusion criteria were defined as follows: (1) holding a valid nursing license and being registered at the participating hospital; (2) ≥ 1 year of clinical nursing experience; (3) providing informed consent. Exclusion criteria were defined as follows: (1) visiting nurses or nursing students not registered at the hospital; (2) nurses absent during the survey period due to maternity, sick, or personal leave.

Of 21,272 distributed questionnaires, 19,729 valid responses were retained (92.75%). Data from 13,323 nurses in 6 cities served as the derivation set (randomly split into training, testing, and internal validation sets at a ratio of 7:2:1). Data from 6406 nurses in the other 3 cities formed an independent external validation set, geographically mutually exclusive with the derivation set.

### 2.2. Ethical Considerations

The study was approved by the Ethics Committee of the Affiliated Hospital of Zunyi Medical University (KLLY‐2025‐001). All participants provided online informed consent.

### 2.3. Variables and Measurements

#### 2.3.1. Outcome Variable

Presenteeism was assessed using the Stanford Presenteeism Scale (SPS‐6; 6 items, two dimensions: completing work and avoiding distractions) [32]. Items are rated on a 5‐point Likert scale (total score 6–30, higher = greater presenteeism). Cronbach’s *α* = 0.700.

#### 2.3.2. Covariates

The 9‐item Utrecht Work Engagement Scale (UWES; three dimensions) [33] was rated on a 7‐point scale; *α* = 0.946. The Psychological Detachment Scale (4 items, 5‐point) [34] had *α* = 0.888. The Perceived Social Support Scale (PSSS; 12 items, three dimensions: family, friend, and significant other support) [35] used a 7‐point scale; *α* = 0.918. The Organizational Climate Questionnaire (17 items, four dimensions: hospital management, department leadership and communication, work collaboration and prospects, doctor–patient understanding, and communication) [36] used a 6‐point scale; *α* = 0.925. The Maslach Burnout Inventory–General Survey (MBI‐GS; 15 items, three dimensions: emotional exhaustion, depersonalization, personal accomplishment) [37] used a 7‐point scale. A composite burnout score was calculated; *α* = 0.907.

A researcher‐designed questionnaire was used to collect information on sociodemographic characteristics (gender, age, marital status, number of children, educational level), work‐related factors (hospital level, working years, professional title, department, monthly income, weekly working hours, night shift involvement, and monthly night shift frequency), and occupational exposures (workplace violence exposure and adverse events in the past 12 months). Workplace violence exposure was defined as self‐reported experience of verbal abuse, threats, or physical violence; adverse events referred to the number of nursing errors or incidents in which the nurse was directly involved or that the nurse reported during the preceding year.

### 2.4. Statistical Analysis

#### 2.4.1. Data Preprocessing

Analyses were performed using SPSS 29.0 (IBM, Armonk, NY) and R 4.4.3 (R Foundation for Statistical Computing, Vienna, Austria). Due to mandatory response checks, no variable had missing values. Continuous variables were tested for normality using the Lilliefors test and presented as mean ± SD or median (Q_1_, Q_3_); group comparisons used an independent‐samples *t*‐test or the Mann–Whitney *U* test. Categorical variables were reported as frequencies (%) and compared with the Pearson chi‐square test. A two‐sided *p* < 0.05 was considered significant. Before modeling, categorical variables were converted to factors. The derivation set (*n* = 13,323) was randomly split at a ratio of 7:2:1 into training, test, and internal validation sets (seed = 123). The external validation set (*n* = 6406) was not involved in any training or selection step.

#### 2.4.2. Predictor Selection

From 20 candidate variables, a two‐stage approach was applied within the training set. First, Least Absolute Shrinkage and Selection Operator (LASSO) with 10‐fold cross‐validation was applied to handle potential collinearity among the 20 candidates through penalized shrinkage (lambda.1se). The confirmatory second step—multivariable linear regression—ensured that retained predictors also demonstrated statistically reliable associations in an unpenalized framework, providing an additional layer of robustness for the downstream interpretability analyses (SHAP, PDP, and network analysis). Variance inflation factor (VIF) was used to assess multicollinearity (threshold > 5). Bootstrap resampling (1000 iterations) evaluated selection stability: variables retained in ≥ 50% of resamples were considered stable.

#### 2.4.3. Machine Learning Risk Estimation Model Construction

Nine regression models were compared: Linear Regression, Elastic Net, Generalized Additive Model (GAM), Random Forest (RF), eXtreme Gradient Boosting (XGBoost), Light Gradient Boosting Machine (LightGBM), Support Vector Regression (SVR), K‐Nearest Neighbors (KNN), and Multilayer Perceptron (MLP). For all models requiring hyperparameter tuning, a 5‐fold cross‐validation grid search was performed. The full search grids and optimal hyperparameters for each model are reported in Supporting Table 1. GAM used thin‐plate regression splines with REML estimation via the mgcv package (v1.9‐1). Scale‐sensitive algorithms (SVR, KNN, MLP) used centered and scaled predictors. Performance was evaluated using root mean square error (RMSE), mean absolute error (MAE), and *R* ^2^, and calibration slopes; 95% CIs for RMSE and MAE were bootstrapped (2000 iterations). Calibration was assessed using slopes and intercepts—the recommended metrics for continuous outcomes—supplemented by calibration plots (Supporting Figure 2).

A sensitivity analysis for clustering effects applied cluster‐robust standard errors with department as the clustering variable to the final linear regression model. The large number of hospitals (*n* = 153) means that any single‐hospital influence on overall estimates is likely limited; the geographically independent external validation provides additional assurance of stability.

SHAP values were computed using the kernelshap (v0.4.1). PDP analyses used the pdp package (v0.8.1) and ggplot2 (v3.5.0). Networks were estimated using qgraph (v1.9.8) with EBICglasso (gamma = 0.5); robustness analyses used bootnet (v1.5.6) and networktools (v1.5.2).

#### 2.4.4. Model Interpretability

Kernel SHAP values were computed with 500 background and 1500 test samples. Mean |SHAP| quantified global importance; beeswarm plots and interaction heatmaps were generated by risk group. Enhanced PDP with percentile markers and density histograms were created for continuous predictors, along with pairwise interaction heatmaps.

#### 2.4.5. Network Analysis

Based on the optimal model, final categorical variables and continuous variable dimensions were included as network nodes within each risk group, with remaining variables as covariates. Global metrics (density and edge counts) and node centrality indices (strength, expected influence, and betweenness) were computed. Robustness was assessed via: (1) nonparametric bootstrap (2000 iterations) for edge weight 95% CIs; (2) case‐dropping bootstrap for correlation stability coefficients (CS‐coefficient > 0.50 indicates acceptable stability); (3) bootstrapped difference tests (*α* = 0.05) for edges and centralities; and (4) EBIC sensitivity analyses with *γ* = 0.25 and *γ* = 0.75.

#### 2.4.6. Sensitivity Analysis for Risk Group Definition

Risk groups were redefined using tertiles of actual SPS‐6 scores. Agreement with predicted‐risk classification was assessed using Cohen’s weighted kappa. SHAP main effects were recalculated within each actual‐risk group.

## 3. Results

### 3.1. Participant Characteristics

Table 1 presents the characteristics of 19,729 nurses. All continuous variables were nonnormally distributed. The majority were female (93.57%), married (78.86%), held a bachelor’s degree or above (87.77%), and worked in tertiary hospitals (66.15%). Most worked 40–45 h/week (56.22%) and were involved in night shifts (INS) (65.58%). Workplace violence and adverse events were reported by 10.47% and 15.11%, respectively.

**TABLE 1:** Comparison of the characteristics of study participants between the derivation set and the external validation set.

Variables	Total (*n* = 19,729)	Derivation set (*n* = 13,323)	External validation set (*n* = 6406)
SPS‐6, M (Q_1_, Q_3_)	14.00 (12.00, 17.00)	14.00 (12.00, 17.00)	14.00 (12.00, 17.00)
Psychological detachment, M (Q_1_, Q_3_)	10.00 (8.00, 13.00)	10.00 (8.00, 13.00)	10.00 (8.00, 13.00)
PSSS, M (Q_1_, Q_3_)	62.00 (50.00, 72.00)	62.00 (50.00, 72.00)	62.00 (50.00, 72.00)
MBI‐GS, M (Q_1_, Q_3_)	1.96 (0.90, 3.00)	2.00 (0.94, 3.00)	1.88 (0.87, 3.00)
UWES, M (Q_1_, Q_3_)	39.00 (25.00, 51.00)	39.00 (24.00, 50.00)	40.00 (26.00, 51.00)
Organizational climate, M (Q_1_, Q_3_)	84.00 (68.00, 97.00)	84.00 (68.00, 96.00)	85.00 (69.00, 98.00)
Gender, *n* (%)			
Male	1268 (6.43)	819 (6.15)	449 (7.01)
Female	18,461 (93.57)	12,504 (93.85)	5957 (92.99)
Age (years), *n* (%)			
≤ 30	6822 (34.58)	4529 (33.99)	2293 (35.79)
31–35	6423 (32.56)	4406 (33.07)	2017 (31.49)
≥ 36	6484 (32.87)	4388 (32.94)	2096 (32.72)
Marital status, *n* (%)			
Unmarried	3572 (18.11)	2348 (17.62)	1224 (19.11)
Married	15,558 (78.86)	10,571 (79.34)	4987 (77.85)
Other	599 (3.04)	404 (3.03)	195 (3.04)
Number of children, *n* (%)			
0	4873 (24.70)	3221 (24.18)	1652 (25.79)
1	6412 (32.50)	4399 (33.02)	2013 (31.42)
≥ 2	8444 (42.80)	5703 (42.81)	2741 (42.79)
Educational level, *n* (%)			
College or below	2412 (12.23)	1504 (11.29)	908 (14.17)
Bachelor’s degree or above	17,317 (87.77)	11,819 (88.71)	5498 (85.83)
Hospital level, *n* (%)			
Secondary	6679 (33.85)	3455 (25.93)	3224 (50.33)
Tertiary	13,050 (66.15)	9868 (74.07)	3182 (49.67)
Working years, *n* (%)			
1–5	4621 (23.42)	3056 (22.94)	1565 (24.43)
6–10	6445 (32.67)	4283 (32.15)	2162 (33.75)
11–15	5464 (27.70)	3784 (28.40)	1680 (26.23)
≥ 16	3199 (16.21)	2200 (16.51)	999 (15.59)
Professional title, *n* (%)			
Junior nurse	1727 (8.75)	1070 (8.03)	657 (10.26)
Senior nurse	9012 (45.68)	6081 (45.64)	2931 (45.75)
Intermediate nurse	7965 (40.37)	5486 (41.18)	2479 (38.70)
Associate chief nurse or above	1025 (5.20)	686 (5.15)	339 (5.29)
Department, *n* (%)			
Internal medicine	5170 (26.21)	3337 (25.05)	1833 (28.61)
Surgery	3807 (19.30)	2553 (19.16)	1254 (19.58)
Obstetrics and gynecology	1750 (8.87)	1242 (9.32)	508 (7.93)
Pediatrics	1542 (7.82)	1124 (8.44)	418 (6.53)
Emergency	1204 (6.10)	840 (6.30)	364 (5.68)
Operating room	961 (4.87)	685 (5.14)	276 (4.31)
Outpatient	925 (4.69)	679 (5.10)	246 (3.84)
Intensive care unit	813 (4.12)	542 (4.07)	271 (4.23)
Others	3557 (18.03)	2321 (17.42)	1236 (19.29)
Monthly income (RMB), *n* (%)			
< 3000	2526 (12.80)	1694 (12.71)	832 (12.99)
3000–5999	12,990 (65.84)	8652 (64.94)	4338 (67.72)
≥ 6000	4213 (21.35)	2977 (22.34)	1236 (19.29)
Weekly working hours, *n* (%)			
< 40 h	2211 (11.21)	1450 (10.88)	761 (11.88)
40–45 h	11,091 (56.22)	7457 (55.97)	3634 (56.73)
≥ 46 h	6427 (32.58)	4416 (33.15)	2011 (31.39)
Involved in night shifts, *n* (%)			
Yes	12,939 (65.58)	8699 (65.29)	4240 (66.19)
No	6790 (34.42)	4624 (34.71)	2166 (33.81)
Night shifts per month, *n* (%)			
0	6790 (34.42)	4624 (34.71)	2166 (33.81)
1–4	3092 (15.67)	1822 (13.68)	1270 (19.83)
5–10	8016 (40.63)	5657 (42.46)	2359 (36.82)
> 10	1831 (9.28)	1220 (9.16)	611 (9.54)
Workplace violence exposure, *n* (%)			
Yes	2066 (10.47)	1522 (11.42)	544 (8.49)
No	17,663 (89.53)	11,801 (88.58)	5862 (91.51)
Adverse events in past 12 months, *n* (%)			
0	16,748 (84.89)	11,189 (83.98)	5559 (86.78)
1‐2	2838 (14.38)	2025 (15.20)	813 (12.69)
≥ 3	143 (0.72)	109 (0.82)	34 (0.53)

*Note:* Continuous variables deviated from normality (assessed by the Lilliefors test) and are presented as median (Q_1_, Q_3_). Categorical variables are presented as frequency (*n*) and percentage (%). Percentages were rounded to the nearest integer. SPS‐6, Stanford Presenteeism Scale; M, Median; Q_1_, First Quartile; Q_3_, Third Quartile.

Abbreviations: MBI‐GS, Maslach Burnout Inventory–General Survey; PSSS, Perceived Social Support Scale; UWES, Utrecht Work Engagement Scale.

### 3.2. Model Development and Validation

#### 3.2.1. Predictor Selection

After LASSO (Supporting Figure 1) and multiple linear regression (Supporting Table 2), five predictors were retained: workplace violence exposure, INS, PSSS, MBI‐GS, and organizational climate. All VIF values were well below 5 (Supporting Table 3). Bootstrap stability analysis (1000 iterations) confirmed robust selection: MBI‐GS (100.0%), PSSS (100.0%), organizational climate (100.0%), workplace violence exposure (91.6%), and INS (91.1%), all exceeding the 50% threshold. Three additional variables (Department, 48.9%; UWES, 48.5%; and adverse events, 45.2%) fell between 25% and 50%; the remaining 12 candidates were below 25% (Supporting Table 4).

#### 3.2.2. Model Construction and Validation

**FIGURE 1:**
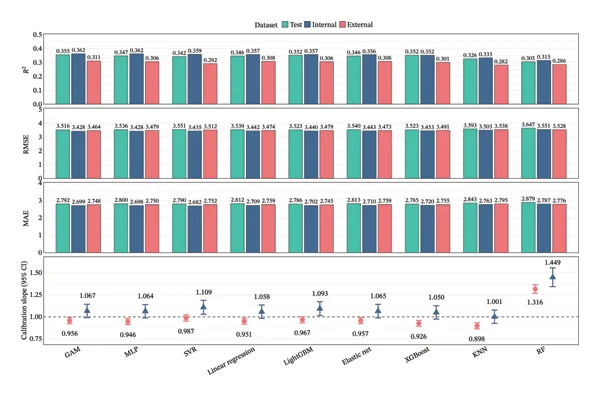
Predictive performance and calibration slopes of nine models across test, internal validation, and external validation datasets. Note: *R* ^2^, RMSE, and MAE for each model on the test (green), internal validation (blue), and external validation (red) datasets. Values are displayed above each bar. Models are ordered by decreasing internal validation *R* ^2^. Calibration slopes with 95% confidence intervals for internal (blue) and external (red) validation. The dashed horizontal line at slope = 1 indicates perfect calibration. Abbreviations: RMSE, root mean square error; MAE, mean absolute error; *R* ^2^, coefficient of determination; Ext, external validation set; GAM, generalized additive model; RF, random forest; XGBoost, eXtreme gradient boosting; LightGBM, light gradient boosting machine; SVR, support vector regression; KNN, K‐nearest neighbors; MLP, multilayer perceptron.

Nine models were developed and externally validated (Figure 1; detailed metrics in Supporting Tables 5 and 6; calibration curves in Supporting Figure 2). In the internal validation set, GAM and MLP both achieved the highest *R* ^2^ (0.362), with comparably low prediction errors (GAM: RMSE = 3.428, MAE = 2.699; MLP: RMSE = 3.428, MAE = 2.698), followed by linear regression (*R* ^2^ = 0.357). GAM’s calibration slopes in internal and external validation were 1.067 (95% CI: 0.991–1.143) and 0.956 (95% CI: 0.921–0.990), both close to 1, with the external slope among the closest of all models. The difference in *R* ^2^ between GAM and linear regression was modest (0.5% points in internal validation; 0.3% points in external validation: 0.311 vs. 0.308); however, GAM offered good calibration and uniquely enabled detection of nonlinear dose–response relationships. The complete GAM specification is provided in Supporting Table 8.

In the cluster‐robust sensitivity analysis, all five predictors retained significance (all *p* < 0.001); standard errors were comparable to the standard model (ratio range: 0.59–1.31; Supporting Table 7).

Based on GAM‐predicted scores, nurses were classified into low‐risk (≤ 12.99), medium‐risk (12.99–15.71), and high‐risk (> 15.71) groups by tertiles. Distributions across all nine models are shown in Supporting Figures 3 and 4.

### 3.3. Model Interpretability

#### 3.3.1. SHAP Analysis

**FIGURE 2:**
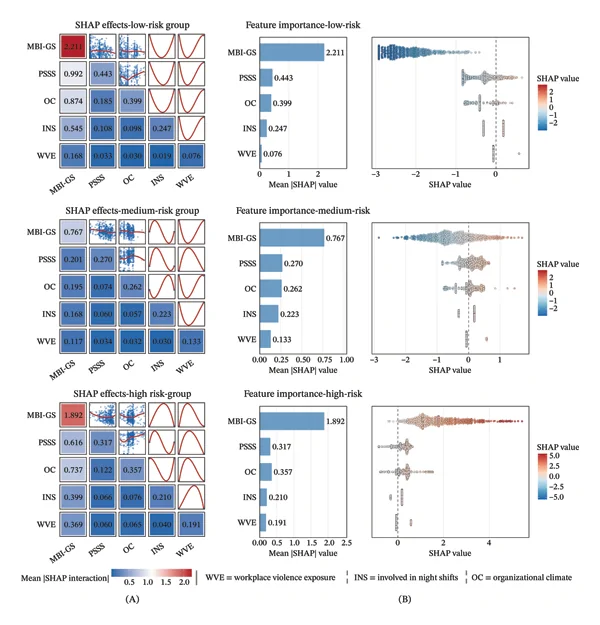
SHAP‐based feature importance, summary plots, and interaction heatmaps across SPS‐6 risk groups. Note: (A) SHAP interaction heatmaps for low, medium, and high risk groups. Diagonal cells show mean |SHAP| (main effect) for each feature; off‐diagonal cells show mean |SHAP_i × SHAP_j| (interaction strength). Color intensity reflects the magnitude of the SHAP interaction, with red indicating stronger interactions. (B) Combined SHAP summary plots. The left panel within each risk group shows feature importance ranked by mean |SHAP|. The right panel shows SHAP beeswarm plots, where each point represents one observation, colored by SHAP value (blue = negative contribution; red = positive contribution to predicted SPS‐6 score). Abbreviations: SHAP, SHapley additive exPlanations; SPS‐6, Stanford presenteeism scale; GAM, generalized additive model; WVE, workplace violence exposure; INS, involved in night shifts; OC, organizational climate; PSSS, perceived social support scale; MBI‐GS, Maslach burnout inventory–general survey.

SHAP main effects and interaction values are presented in Figure 2 (full values in Supporting Tables 9 and 10). Across all risk levels, MBI‐GS had the highest main effect, with a “high at both ends, low in the middle” pattern (low: 2.211; medium: 0.767; high: 1.892). PSSS and organizational climate showed a similar pattern. Workplace violence exposure increased progressively with risk level (0.076–0.133–0.191). INS fluctuated minimally (0.210–0.247).

The top five interaction pairs in each group were all centered on MBI‐GS. The MBI‐GS × PSSS interaction was strongest in the low‐risk group (0.992), while MBI‐GS × organizational climate predominated in the high‐risk group (0.737). All interaction intensities were attenuated in the medium‐risk group (≤ 0.201). The workplace violence × MBI‐GS interaction showed a U‐shaped pattern across risk groups (0.168 in low‐risk, 0.117 in medium‐risk, and 0.369 in high‐risk), with a marked increase from medium to high risk. PSSS ranked second and organizational climate third in low‐ and medium‐risk groups, but their order reversed in the high‐risk group.

#### 3.3.2. PDP Analysis

**FIGURE 3:**
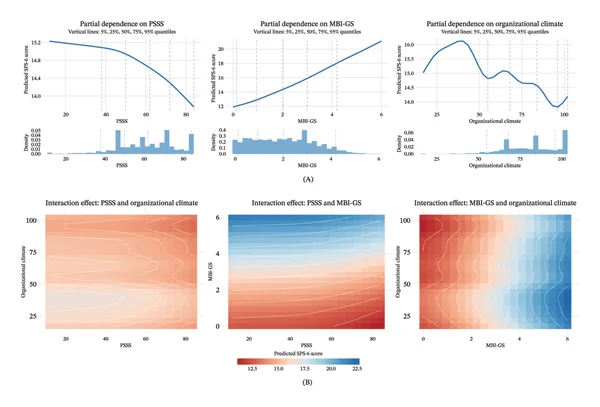
Partial dependence plots for continuous predictors and their two‐way interactions from the GAM model. Note: (A) Enhanced partial dependence plots for the three continuous core predictors (PSSS, MBI‐GS, organizational climate). The upper panel of each plot shows the partial dependence curve—the expected predicted SPS‐6 score as a function of the focal predictor, averaged over the distribution of all other predictors. Vertical dashed lines mark the 5th, 25th, 50th, 75th, and 95th percentiles of the predictor’s distribution in the training set. The lower panel shows the density distribution of each predictor. (B) Two‐way interaction PDP heatmaps for the three pairs of continuous predictors. Color intensity indicates the predicted SPS‐6 score, with warmer colors (red) representing higher predicted scores and cooler colors (blue) representing lower predicted scores. White contour lines delineate isoprediction regions. All predictions were generated from the final GAM model. Abbreviations: PDP, partial dependence plot; GAM, generalized additive model; SPS‐6, Stanford presenteeism scale; PSSS, perceived social support scale; MBI‐GS, Maslach burnout inventory–general survey.

Figure 3 presents PDP curves (detailed values in Supporting Tables 11 and 12). PSSS exhibited a gently descending trend, with the decline accelerating beyond a score of approximately 50 (overall change ∼1.5 points). MBI‐GS rose approximately linearly (from 11.90 at score 0 to 21.13 at score 6, a range of > 9 points)—the largest influence among all predictors. Organizational climate showed an inverted U‐shape peaking at approximately 40 points, followed by a wave‐like decline.

Pairwise interaction PDPs revealed that the MBI‐GS × PSSS combination with high burnout and low social support yielded the highest predicted score (21.81), whereas the combination in which both were low gave the lowest (11.09; range = 10.71). The MBI‐GS × organizational climate interaction showed a similar span (range = 11.49), with the moderate‐organizational‐climate and high‐burnout combination producing lower predictions than extreme combinations. The PSSS × organizational climate interaction had the smallest range (3.74).

### 3.4. Network Analysis

**FIGURE 4:**
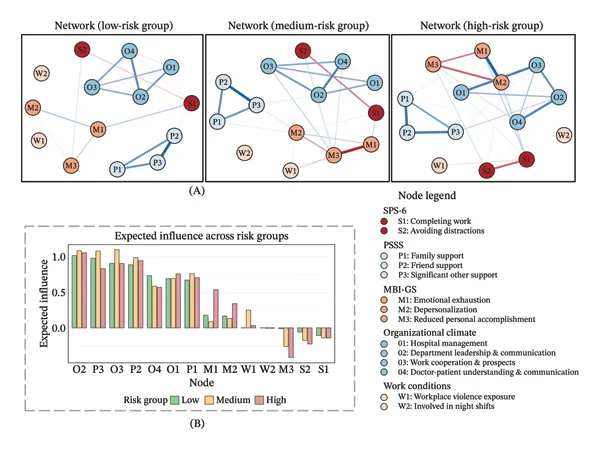
Regularized partial correlation networks and node expected influence across SPS‐6 risk groups. Note: (A) Regularized partial correlation networks for low, medium, and high SPS‐6 risk groups. Each network contains 14 nodes representing the five core predictors and nine subscale‐level items. Nodes are colored by variable domain: red = SPS‐6, light blue = PSSS, orange = MBI‐GS, medium blue = organizational climate, beige = work conditions. Blue edges denote positive regularized partial correlations; red edges denote negative regularized partial correlations. Edge thickness reflects the magnitude of the association. Networks were estimated using EBICglasso (gamma = 0.5) on residuals after regressing out demographic and work‐related covariates. (B) Grouped bar plot of expected influence for each node across the three risk groups. Nodes are ordered by descending EI in the low‐risk group. EI > 0 indicates a net positive influence on the network; EI < 0 indicates a net negative influence. Abbreviations: SPS‐6, Stanford presenteeism scale; PSSS, perceived social support scale; MBI‐GS, Maslach burnout inventory–general survey; GAM, generalized additive model; W1, workplace violence exposure; W2, involved in night shifts; S1, completing work; S2, avoiding distractions; P1, family support; P2, friend support; P3, significant other support; M1, emotional exhaustion; M2, depersonalization; M3, reduced personal accomplishment; O1, hospital management; O2, department leadership and communication; O3, work cooperation and prospects; O4, doctor–patient understanding and communication.

Regularized partial correlation networks were constructed for each risk group (14 nodes; Figure 4; global metrics in Supporting Table 13). Robustness diagnostics supported stability: edge weight 95% CIs were narrow (Supporting Figure 5); CS‐coefficients for strength (0.59–0.67) and expected influence (0.63–0.71) all exceeded the 0.50 threshold (Supporting Figure 6); core nodes showed significantly higher centrality than peripheral nodes (*p* < 0.05; Supporting Figure 7); and EBIC sensitivity analyses with *γ* = 0.25 and 0.75 preserved the density gradient (Supporting Figure 8).

Network connectivity became denser with increasing risk: nonzero edges rose from 50 (low) to 61 (medium) to 68 (high), with densities of 0.549, 0.670, and 0.747. Negative edges increased from 20 in low/medium to 31 in high‐risk.

O2 (department leadership and communication), O3 (work collaboration and prospects), and P3 (significant other support) were stable core nodes across all risk groups, with consistently high positive expected influence (EI). MBI‐GS dimensions showed marked risk‐dependent centrality enhancement: M1 (emotional exhaustion) strength rose from 0.54 (low) to 1.25 (high); M2 (depersonalization) from 0.33 to 1.12; M1 and M2 EI surged in the high‐risk group (to 0.53 and 0.34). M3 (reduced personal accomplishment) had negative EI across all groups, increasingly negative with risk escalation (−0.01 to −0.26 to −0.42). Workplace violence exposure (W1) was nearly peripheral in the low‐risk group but gained centrality in medium/high‐risk groups, showing bridging connections with burnout and organizational climate nodes. INS (W2) contributed minimal direct influence (strength < 0.04 in all groups). The two SPS‐6 nodes (S1, S2) showed negative EI across all groups, consistent with their outcome positioning. Node EI values are detailed in Supporting Table 14.

### 3.5. Sensitivity Analysis: Actual SPS‐6 Tertiles

Agreement between predicted and actual tertile classification was moderate (weighted kappa = 0.54), with confusion primarily between adjacent categories. SHAP feature importance patterns were highly consistent with the primary analysis (Supporting Table 15): MBI‐GS remained dominant (1.665, 1.345, 1.726 across low‐, medium‐, and high‐actual‐risk groups), workplace violence exposure showed progressive increase (0.108, 0.114, 0.184), and PSSS/organizational climate maintained their rank order (Supporting Figure 9). The more pronounced “high at both ends” pattern in predicted‐risk groups is expected: when groups are defined by model predictions, the dominant feature exhibits maximum contrast; observed‐score grouping naturally attenuates this contrast because each group contains a mixture of model‐classified risk levels. Critically, the qualitative pattern of feature importance was preserved across both schemes.

## 4. Discussion

### 4.1. Performance and Rationale for GAM Selection

To our knowledge, this is the first study to develop and externally validate machine learning‐based risk estimation models for clinical nurse presenteeism using multicenter data (19,729 nurses, 9 cities). Among nine algorithms with hyperparameter tuning, GAM was selected for its balanced performance: the highest internal validation *R* ^2^ (0.362, tied with MLP), a good internal calibration slope (1.067, close to 1), an external calibration slope (0.956) second only to Elastic Net (0.957) among models with comparable *R* ^2^, and the capacity to capture nonlinear relationships.

We acknowledge that the gain in *R* ^2^ over linear regression was modest (0.5% points), and it is plausible that the principal conclusions—particularly the primacy of burnout and the protective role of social support—could have been obtained through more parsimonious linear approaches. Furthermore, in external validation the model explained approximately 31% of the variance (*R* ^2^ = 0.311), leaving roughly 69% of variability in presenteeism unexplained. GAM’s selection was, therefore, driven not by a substantial improvement in global variance explained, but by three considerations beyond global *R* ^2^: (1) good calibration, with both internal and external slopes close to 1 and the external slope being the second‐best among all models (0.956, after Elastic Net’s 0.957); (2) the capacity to fit nonlinear smooth terms, revealing clinically meaningful patterns—most notably the inverted U‐shaped organizational climate association—entirely obscured in linear models; and (3) additive interpretability facilitating SHAP, PDP, and network analyses. Wu et al. [20] constructed a nomogram for ICU nurse presenteeism without independent external validation; the present GAM is thus among the first independently externally validated risk estimation models in this field, though its clinical utility for guiding interventions remains to be established through prospective studies. Consistent with prior research [21, 23, 25], the principal advantage of machine learning may lie not in large gains in global variance explained but in revealing heterogeneous, nonlinear effect patterns that directly inform stratified interventions.

### 4.2. Patterns of Predictor Influence

#### 4.2.1. SHAP Findings

SHAP analysis identified MBI‐GS as the strongest predictor across all risk levels, with contribution most pronounced at extremes—consistent with prior evidence linking burnout to presenteeism [7, 9, 24]. From a Conservation of Resources (COR) theory perspective, burnout reflects continuous resource depletion impairing work engagement [7, 9].

PSSS and organizational climate exhibited a “high at both ends, low in the middle” pattern, suggesting their protective effects are more pronounced in extreme risk contexts. Previous studies confirm the buffering role of social support [17, 18, 20]; our findings further indicate that this buffering is not uniformly distributed. The mechanism through which organizational climate operates may be more complex than linear mediation models suggest [38, 39].

SHAP interaction analysis revealed risk‐context–dependent patterns: MBI‐GS × PSSS dominated in the low‐risk group (social support buffering burnout before resource depletion becomes severe), whereas MBI‐GS × organizational climate predominated in the high‐risk group (systematic organizational support becoming critical when individual‐level resources are insufficient). The workplace violence × MBI‐GS interaction intensified markedly in the high‐risk group (0.369), consistent with the chain mediation pathway identified by Li and Fu [40], suggesting a synergistic association in extreme risk contexts, though this does not imply direct causation. It should be noted that SHAP importance rankings reflect variable contributions within the specific modeling framework and the particular set of predictors retained, and do not independently establish intervention priority. Variables not measured in this study—such as personality traits, sleep quality, or objective workload—might exhibit different importance patterns if included. Similarly, the dominant contribution of MBI‐GS partly reflects its conceptual proximity to presenteeism as a psychological outcome; other types of predictors might show stronger associations with different operationalizations of productivity loss. SHAP findings are, therefore, best viewed as hypothesis‐generating for future intervention research rather than as direct guidance for resource allocation.

#### 4.2.2. Nonlinear Dose–Response Relationships and Synergistic Patterns Based on PDP

PDP revealed that MBI‐GS had an approximately linear relationship with SPS‐6 and the largest range of influence (> 9 points), consistent with multiple prior studies [7–9]. PSSS showed a gently descending trend with an accelerated decline beyond a score of ∼50, suggesting a threshold at which social support’s protective effect becomes substantially enhanced [17, 18, 28].

Organizational climate exhibited a nonlinear trajectory: an inverted U‐shape peaking at ∼40 points followed by a wave‐like decline. Drawing on the Job Demands–Resources (JD‐R) model, one possible interpretation is that moderate organizational climate may coincide with a transitional period of demand–resource mismatch, where management expectations increase alongside emerging but not‐yet‐mature support structures [41–43]. However, we emphasize that this mechanistic interpretation is speculative; constructs such as “adaptation pressure” were not directly measured, and the observed curve may reflect unmeasured confounders. Future longitudinal studies with direct measurement of these proposed mediating constructs are needed to empirically test these hypotheses.

Pairwise PDP interactions confirmed that SPS‐6 is likely to result from nonlinear synergistic effects among core variables, not simple additive accumulation—explaining why GAM’s ability to fit complex nonlinear relationships proved advantageous [21, 23, 25].

### 4.3. Network Structure and Core Nodes

Network analysis revealed risk‐dependent structural reorganization: denser connectivity and more negative edges under high risk, consistent with findings of strong interconnectedness between job demands–resources and depressive symptoms in ICU nurses [28]. This suggests that risk escalation involves intensified coupling among psychosocial elements, where disturbance in one node may propagate rapidly.

O2 (department leadership and communication), O3 (work collaboration and prospects), and P3 (significant other support) were stable protective cores—aligning with Li et al. [28], who identified supervisor support and coworker support as nodes with the highest EI among job resources. MBI‐GS centrality showed risk‐dependent enhancement, extending findings by Zhang et al. [31] on age differences in burnout centrality. Workplace violence exhibited a bridging role during risk escalation, consistent with the violence–burnout–productivity impairment pathway [15]. Night shifts contributed limited direct network influence, though prior research confirms their independent predictive role [10], likely operating through physiological mechanisms (fatigue, sleep disruption) [8, 10, 17] rather than the psychosocial network assessed here. As with the SHAP analyses, network centrality indices are conditional on the set of nodes included and the estimation algorithm employed. The identification of department leadership and communication (O2) as a core node suggests a potentially important role that warrants further investigation, but does not in itself establish that interventions targeting this node would yield greater benefits than interventions targeting more peripheral nodes.

### 4.4. Hypotheses for Future Intervention Research

Although the cross‐sectional design precludes direct intervention recommendations, the observed patterns suggest several hypotheses that could be tested in future prospective implementation research:

For nurses with low estimated risk, the prominent MBI‐GS × PSSS interaction observed at this level suggests that early social support enhancement (e.g., peer mentoring, coworker networks) may be worth evaluating as a preventive resource‐building strategy. Whether strengthening social support before burnout becomes severe can reduce subsequent presenteeism remains to be tested longitudinally.

For nurses with medium estimated risk, the PDP‐identified thresholds—PSSS declining more steeply beyond approximately 50 and organizational climate peaking near 40—suggest candidate screening indicators that could be examined for their prospective predictive value. Future studies could evaluate whether monitoring these indicators helps identify nurses approaching meaningful risk transitions.

For nurses with high estimated risk, the network findings—increased burnout centrality, denser negative connectivity, and the bridging role of workplace violence—suggest that multicomponent organizational interventions (e.g., strengthening department leadership and communication, improving career development pathways, and establishing violence‐reporting procedures with timely psychological support) may warrant evaluation as bundled strategies. Whether such interventions can interrupt the synergistic associations observed among burnout, workplace violence, and presenteeism at high risk levels is a question for future interventional studies.

### 4.5. Strengths and Limitations

Key strengths include the following: (1) systematic comparison of nine machine learning algorithms with hyperparameter tuning and geographically independent external validation; (2) integration of SHAP, PDP, and risk‐stratified network analysis with formal robustness diagnostics; (3) detection of nonlinear patterns inaccessible to linear models; and (4) bootstrap‐confirmed predictor selection stability (all > 90%).

Several limitations should be noted. First, the cross‐sectional design limits causal inference and means the model estimates concurrent rather than prospective risk. Second, the sample was drawn from a single Chinese province; generalizability to other regions requires further examination. Third, the model explained ∼31% of variance externally, leaving ∼69% unexplained—comparable to or higher than related cross‐sectional nursing prediction studies [21, 22]. Unmeasured domains include individual factors (personality, sleep quality), job‐level factors (nurse‐to‐patient ratios, objective workload), unit‐level factors (team cohesion, staffing adequacy), and contextual factors (work‐family conflict). Fourth, participants were nested within departments across 153 hospitals; however, cluster‐robust sensitivity analysis showed no meaningful impact on inference. Future multilevel modeling should partition variance across individual, department, and hospital levels. Fifth, the SPS‐6 reliability (*α* = 0.700) was acceptable but lower than that of other scales, partly attributable to its brevity (6 items, two dimensions); this value is consistent with prior Chinese nurse studies (0.68–0.74) and exceeds the 0.70 threshold, though measurement error may have attenuated model fit. Sixth, candidate variables were limited to self‐reported psychosocial and work‐related factors. Seventh, risk‐stratified network comparisons involve inherent circularity, as risk groups are model‐derived functions of the same variables analyzed in the networks. Observed structural differences may partly reflect this dependency. Network comparisons should, therefore, be viewed as exploratory descriptions across model‐defined risk levels rather than independent evidence of causal group‐level reorganization. Future studies could mitigate this by defining groups using an external criterion.

## 5. Conclusion

Based on multicenter data from 19,729 clinical nurses, this study developed and externally validated risk estimation models using nine machine learning algorithms. Five independent predictors were identified (MBI‐GS, PSSS, organizational climate, workplace violence exposure, and INS), all with bootstrap selection frequencies > 90%. GAM was selected for its balanced performance and capacity to capture nonlinear relationships. Through SHAP, PDP, and risk‐stratified network analysis with formal robustness diagnostics, this study delineated nonlinear dose–response relationships, risk‐dependent interaction patterns, and dynamic shifts in psychosocial variable association structures. Burnout was the strongest predictor, with its contribution and network centrality most pronounced in extreme risk groups; department leadership/communication and work collaboration served as stable protective nodes. These findings, while derived from cross‐sectional data, provide preliminary evidence that may inform the design of prospective studies and the development of risk‐stratified intervention strategies.

## Author Contributions

Yumeng Zhang: conceptualization, methodology, validation, formal analysis, data curation, writing–original draft, writing–review and editing, and visualization. Shaoting Yang: methodology, formal analysis, data curation, and writing–review and editing. Qingfen Zeng: investigation, validation, and writing–review and editing. Honghong Wen: investigation, data curation, and writing–review and editing. Heting Liang: investigation, validation, and writing–review and editing. Yunting Li: data curation, investigation, and writing–review and editing. Shiming Huang: data curation, resources, and writing–review and editing. Huiming Gao: writing–review and editing. Chaoping Wang: project administration and writing–review and editing. Xiaoli Yuan: conceptualization, methodology, supervision, project administration, funding acquisition, and writing–review and editing.

## Funding

This study was supported by the Guizhou Provincial Science and Technology Planning Project (Qian Ke He Zhi Cheng [2020]4Y174) and the Project of Guizhou Provincial Nursing Association (GZHLKY202505).

## Conflicts of Interest

The authors declare no conflicts of interest.

## Supporting Information

Additional supporting information can be found online in the Supporting Information section.

## Supporting information


**Supporting Information 1** Supporting File 1: PDF file containing Supporting Figures 1–9 and Supporting Tables 1–15. Supporting Table 1: Hyperparameter tuning summary. Reports the search ranges and final selected values for all hyperparameters across the nine candidate models. Supporting Figure 1: LASSO feature selection. Upper panel displays coefficient paths against the L1 norm (lambda), with each curve representing a predictor’s coefficient shrinking toward zero. Lower panel shows the 10‐fold cross‐validated mean squared error (MSE) curve; the left dashed line indicates lambda.min, and the right dashed line indicates lambda.1se, which retained five predictors with nonzero coefficients. Supporting Table 2: Multivariable linear regression results in the training set for the five predictors selected by LASSO, including unstandardized coefficients, standard errors, *t*‐values, *p* values, and 95% confidence intervals. Supporting Table 3: Variance inflation factors (VIF) for the five final predictors, confirming the absence of serious multicollinearity (all VIF < 5). Supporting Table 4: Bootstrap variable selection stability. Summarizes the selection frequency of each candidate predictor across 1000 bootstrap resamples, demonstrating the robustness of the final variable set. Supporting Table 5: Model predictive performance across datasets. Provides RMSE, MAE, *R* ^2^, and adjusted *R* ^2^ for all nine models in the test, internal validation, and external validation datasets. Supporting Table 6: Model calibration slopes and intercepts across datasets. Reports calibration slopes and intercepts with 95% confidence intervals for all models in both internal and external validation datasets. Supporting Figure 2: Calibration plots for all models in the internal validation set, comparing predicted versus observed SPS‐6 scores against the ideal 45‐degree line. Supporting Figure 3: Risk stratification violin plots across all nine models in the internal validation set, showing the distribution of actual SPS‐6 scores within low‐, medium‐, and high‐risk groups defined by tertiles of each model’s predicted scores. Supporting Figure 4: Risk stratification scatter plots for all nine models in the internal validation set, depicting the correspondence between predicted and actual SPS‐6 scores within each risk group. Supporting Table 7: Cluster‐robust standard errors (department‐level clustering). Presents regression coefficients and cluster‐robust standard errors to account for clustering of participants within departments. Supporting Table 8: GAM model specification and coefficient estimates. Details the effective degrees of freedom, smooth terms, and parametric coefficient estimates for the generalized additive model. Supporting Table 9: Top five SHAP interaction pairs for each risk group. Ranks the strongest pairwise SHAP interactions (by mean |SHAP_i × SHAP_j|) within low, medium, and high predicted risk strata. Supporting Table 10: Mean absolute SHAP values for five core predictors by risk group. Quantifies the average absolute SHAP contribution of each predictor across the three risk groups. Supporting Table 11: Partial dependence values at key percentiles of continuous predictors. Shows predicted SPS‐6 scores at the minimum, 5th, 25th, 50th, 75th, 95th, and maximum of the three continuous predictors (PSSS, MBI‐GS, and organizational climate). Supporting Table 12: Summary of two‐way interaction partial dependence predictions from the GAM model. Reports the minimum, maximum, and range of predicted SPS‐6 scores for each pairwise interaction among the three continuous predictors. Supporting Table 13: Global metrics of risk‐stratified networks. Lists total nodes, total possible edges, nonzero edges, density, and counts of positive and negative edges for the regularized partial correlation networks estimated within each SPS‐6 risk group. Supporting Table 14: Node‐level centrality indices by risk group. Provides strength, expected influence, and betweenness centrality for all 14 network nodes across low‐, medium‐, and high‐risk networks. Supporting Figure 5: Bootstrapped edge weight 95% confidence intervals for each risk group network, illustrating the stability of estimated partial correlations. Supporting Figure 6: Case‐dropping bootstrap CS‐coefficient plots, illustrating the stability of node centrality indices as cases are progressively removed. Supporting Figure 7: Bootstrapped difference tests for edge weights and node strengths, displaying statistically significant differences in network connections and node strength between risk groups. Supporting Figure 8: Sensitivity networks estimated with EBIC tuning parameters *γ* = 0.25, 0.50, and 0.75, demonstrating the robustness of network structure to the choice of the tuning parameter. Supporting Table 15: SHAP main effects by actual SPS‐6 risk group. Presents mean SHAP values for each predictor when participants are grouped by their observed (actual) SPS‐6 tertiles. Supporting Figure 9: SHAP feature importance: predicted versus actual SPS‐6 tertiles. Compares SHAP‐based feature importance rankings between risk groups defined by predicted SPS‐6 scores and those defined by actual SPS‐6 scores.

**Supporting Information 2** STROBE‐checklist‐v4‐cross‐sectional.

## Data Availability

The data that support the findings of this study are available on request from the corresponding author. The data are not publicly available due to privacy or ethical restrictions.
