# Supplementary material for: Development and External Validation of a Machine Learning Model for Risk Stratification of Presenteeism in Clinical Nurses: A Multicenter Cross‐Sectional Study
Source: J Nurs Manag. 2026 Sep 9;2026:6257117. doi: 10.1155/jonm/6257117 (PMC13559007; doi:10.1155/jonm/6257117)

Supplementary File

Contents

Supplementary Table 1 Hyperparameter tuning summary 1
Supplementary Figure 1 LASSO feature selection 2
Supplementary Table 2 Multivariable linear regression results (training set) 3
Supplementary Table 3 VIF for final model predictors 4
Supplementary Table 4 Bootstrap variable selection stability 5
Supplementary Table 5 Model predictive performance across datasets 6
Supplementary Table 6 Model calibration slopes and intercepts across datasets 7
Supplementary Figure 2 Calibration plots for all models (internal validation set) 8
Supplementary Figure 3 Risk stratification violin plots across models (internal validation set) 9
Supplementary Figure 4 Risk stratification scatter plots for all models (internal validation set) 10
Supplementary Table 7 Cluster-robust standard errors (department-level clustering) 11
Supplementary Table 8 GAM model specification and coefficient estimates 12
Supplementary Table 9 Top five SHAP interaction pairs for each risk group 13
Supplementary Table 10 Mean absolute SHAP values for five core predictors by risk group 14
Supplementary Table 11 Partial dependence values at key percentiles of continuous predictors 15
Supplementary Table 12 Summary of two-way interaction partial dependence predictions from the GAM model 16
Supplementary Table 13 Global metrics of risk-stratified networks 17
Supplementary Table 14 Node-level centrality indices by risk group 18
Supplementary Figure 5 Bootstrapped edge weight 95% CIs for each risk group network 20
Supplementary Figure 6 Case-dropping bootstrap CS-coefficient plots 21
Supplementary Figure 7 Bootstrapped difference tests for edge weights and node strengths 22
Supplementary Figure 8 Sensitivity networks with EBIC tuning parameters γ = 0.25, 0.50, and 0.75 23
Supplementary Table 15 SHAP main effects by actual SPS-6 risk group 24
Supplementary Figure 9 SHAP feature importance: predicted vs. actual SPS-6 tertiles25

Supplementary Table 1 Hyperparameter tuning summary

Model	Search Grid	Optimal Hyperparameters	Tuning Method
Linear Regression	—	No hyperparameters	—
Elastic Net	α = {0, 0.1, 0.2, 0.3, 0.4, 0.5, 0.6, 0.7, 0.8, 0.9, 1.0}; λ = 10^seq(–3, 1, length = 50)	α = 0.10, λ = 0.02947	5-fold CV grid search
GAM	— (smooth terms automatically selected)	s(PSSS) edf = 2.92, s(MBI-GS) edf = 3.73, s(Organizational Climate) edf = 7.23; method = REML	GCV (automatic via mgcv)
Random Forest	mtry = {1, 2, 3, 4, 5}; ntree = 500	mtry = 2, ntree = 500	5-fold CV grid search
XGBoost	nrounds = {50, 100, 150, 200}; max_depth = {2, 3, 4, 5}; eta = {0.01, 0.05, 0.10, 0.20}; subsample = {0.6, 0.8, 1.0}; colsample_bytree = {0.6, 0.8, 1.0}	nrounds = 100, max_depth = 3, eta = 0.05, subsample = 0.8, colsample_bytree = 0.8	5-fold CV grid search
LightGBM	num_leaves = {7, 15, 31, 63}; learning_rate = {0.01, 0.05, 0.10}; nrounds = {20, 50, 100}; feature_fraction = {0.6, 0.8, 1.0}	num_leaves = 15, learning_rate = 0.10, nrounds = 37, feature_fraction = 0.8	5-fold CV grid search
SVR	kernel = radial; C = {0.01, 0.1, 1, 10, 100}; sigma = {0.001, 0.01, 0.1, 1}	kernel = radial, C = 10.00, sigma = 0.01	5-fold CV grid search
KNN	k = {5, 10, 15, 20, 30, 50, 70, 100}	k = 50	5-fold CV grid search
MLP	size = {1, 2, 3, 5, 7, 10}; decay = {0, 0.01, 0.10}	hidden = 1 layer, size = 3, decay = 0.10	5-fold CV grid search

Notes: All models requiring hyperparameter tuning (Elastic Net, Random Forest, XGBoost, LightGBM, SVR, KNN, MLP) were tuned via a 5-fold cross-validation grid search within the training set, with the search grids specified in the table. For Elastic Net, λ was searched over 50 log-linearly spaced values between 10^{-3} and 10^{1}. GAM smooth terms and effective degrees of freedom were automatically estimated via generalized cross-validation with restricted maximum likelihood (REML) using the mgcv package (v1.9-1). Linear Regression has no hyperparameters. All analyses were performed in R 4.4.3. For LightGBM, early stopping with 10 rounds of patience was applied within each cross-validation fold; nrounds = 37 represents the optimal stopping iteration, not a value directly searched in the grid.

Abbreviations: CV, cross-validation; GCV, generalized cross-validation; REML, restricted maximum likelihood; edf, effective degrees of freedom.

Supplementary Figure 1 LASSO feature selection

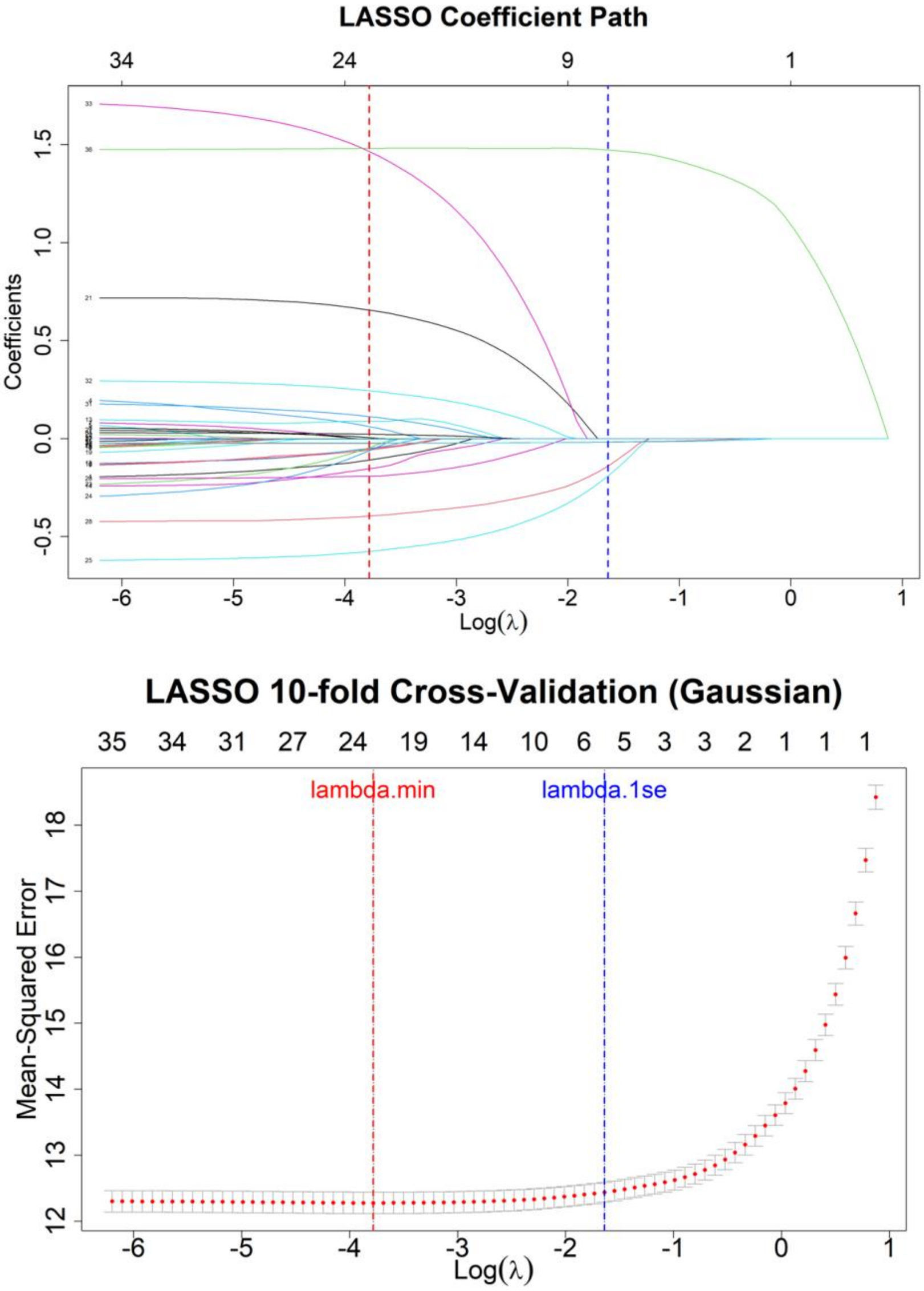

Notes: LASSO feature selection. Upper panel: coefficient paths against the L1 norm (lambda). Each curve represents a predictor's coefficient, shrinking toward zero as lambda increases. Lower panel: 10-fold cross-validated mean squared error (MSE) curve. The left vertical dashed line (red) marks lambda.min, the lambda giving minimum MSE; the right vertical dashed line (blue) marks lambda.1se, the largest lambda within one standard error of the minimum. The five predictors with non-zero coefficients at lambda.1se were retained.

Abbreviations: LASSO, Least Absolute Shrinkage and Selection Operator; MSE, Mean Squared Error.

Supplementary Table 2 Multivariable linear regression results (training set)

Variables	β	S.E	t	*P*	β (95%CI)
Intercept	15.11	0.26	59.22	<0.001	15.11 (14.61 ~ 15.61)
Workplace violence exposure					
Yes					0.00 (Reference)
No	-0.70	0.10	-7.09	<0.001	-0.70 (-0.89 ~ -0.51)
Involved in night shifts					
Yes					0.00 (Reference)
No	-0.45	0.06	-6.96	<0.001	-0.45 (-0.58 ~ -0.32)
PSSS	-0.02	0.00	-9.02	<0.001	-0.02 (-0.03 ~ -0.02)
MBI-GS	1.59	0.03	53.47	<0.001	1.59 (1.53 ~ 1.65)
Organizational Climate	-0.02	0.00	-7.62	<0.001	-0.02 (-0.02 ~ -0.01)

Notes: β = unstandardized regression coefficient; S.E. = standard error; CI = confidence interval.

Abbreviations: PSSS, Perceived social support scale; MBI-GS, Maslach Burnout Inventory-General Survey.

Supplementary Table 3 VIF for final model predictors

Variable	VIF
Workplace violence exposure	1.064
Involved in night shifts	1.016
PSSS	1.565
MBI-GS	1.565
Organizational Climate	1.763

Note: VIF values < 5 indicate no serious multicollinearity among predictors.

Abbreviations: VIF, Variance inflation factors; PSSS, Perceived social support scale; MBI-GS, Maslach Burnout Inventory-General Survey.

Supplementary Table 4 Bootstrap variable selection stability

Variable	Selection Frequency (%)
PSSS	100
MBI-GS	100
Organizational Climate	100
Workplace violence exposure	91.6
Involved in night shifts	91.1
Department	48.9
UWES	48.5
Number of adverse events in the past 12 months	45.2
Weekly working hours	24.4
Night shifts per month	6.2
Professional title	4.5
Gender	2.8
Psychological Detachment	2.7
Monthly income RMB	0.3
Working years	0.2
Age years	0.1
Marital status	0.1
Educational level	0.1
Hospital level	0.1
Number of children	0

Notes: Selection frequencies from 1,000 bootstrap resamples of the training set. In each resample, LASSO with lambda.1se was applied. Variables with frequency ≥ 50% were considered stable. The five final predictors (boldfaced) all exceeded this threshold.

Supplementary Table 5 Model predictive performance across datasets

Model	Test RMSE	Test MAE	Test R^2	Valid RMSE (95% CI)	Valid MAE (95% CI)	Valid R^2 (95% CI)	Adjusted R^2	Ext RMSE	Ext MAE	Ext R^2
Linear Regression	3.539	2.812	0.346	3.442 (3.298–3.588)	2.709 (2.595–2.825)	0.357 (0.310–0.396)	0.332	3.474	2.759	0.308
Elastic Net	3.540	2.813	0.346	3.443	2.71	0.356	0.332	3.473	2.759	0.308
**GAM**	**3.516**	**2.792**	**0.355**	**3.428 (3.287–3.570)**	**2.699 (2.587–2.811)**	**0.362 (0.319–0.401)**	**0.336**	**3.464**	**2.748**	**0.311**
RF	3.647	2.879	0.305	3.551	2.787	0.315	—	3.528	2.776	0.286
XGBoost	3.523	2.785	0.352	3.453	2.72	0.352	—	3.491	2.755	0.301
LightGBM	3.523	2.786	0.352	3.44	2.702	0.357	—	3.479	2.745	0.306
SVR	3.551	2.79	0.342	3.435	2.682	0.359	—	3.512	2.752	0.292
KNN	3.593	2.843	0.326	3.503	2.763	0.333	—	3.538	2.795	0.282
MLP	3.536	2.8	0.347	3.428	2.698	0.362	—	3.479	2.75	0.306

Notes: 95% confidence intervals (in parentheses) were estimated via bootstrap resampling (2,000 iterations) of the internal validation set and are reported for the two primary models (GAM and Linear Regression). Adjusted R^2 is reported only for models with a closed-form solution (Linear Regression, Elastic Net, GAM). "—" indicates not applicable for tree-based, kernel-based, and neural network models. GAM was selected as the final model.

Abbreviations: RMSE, root mean square error; MAE, mean absolute error; R^2, coefficient of determination; CI, confidence interval; Ext, external validation set; GAM, Generalized Additive Model; RF, Random Forest; XGBoost, eXtreme Gradient Boosting; LightGBM, Light Gradient Boosting Machine; SVR, Support Vector Regression; KNN, K-Nearest Neighbors; MLP, Multi-Layer Perceptron.

Supplementary Table 6 Model calibration slopes and intercepts across datasets

Model	Internal Slope (95% CI)	Internal Intercept (95% CI)	External Slope (95% CI)	External Intercept (95% CI)
Linear Regression	1.058 (0.982-1.135)	-0.772 (-1.892-0.349)	0.951 (0.916-0.985)	0.513 (0.007-1.019)
Elastic Net	1.065 (0.988-1.142)	-0.865 (-1.993-0.262)	0.957 (0.922-0.992)	0.426 (-0.084-0.935)
GAM	1.067 (0.991-1.143)	-0.894 (-2.010-0.221)	0.956 (0.921-0.990)	0.433 (-0.072-0.938)
RF	1.449 (1.343-1.556)	-6.476 (-8.036- -4.917)	1.316 (1.267-1.364)	-4.814 (-5.518- -4.110)
XGBoost	1.050 (0.974-1.127)	-0.594 (-1.711-0.523)	0.926 (0.891-0.960)	0.901 (0.402-1.400)
LightGBM	1.093 (1.015-1.172)	-1.281 (-2.432- -0.129)	0.967 (0.931-1.002)	0.268 (-0.250-0.785)
SVR	1.109 (1.030-1.187)	-1.856 (-3.033- -0.679)	0.987 (0.951-1.023)	-0.331 (-0.871-0.208)
KNN	1.001 (0.925-1.077)	0.026 (-1.096-1.148)	0.898 (0.864-0.933)	1.223 (0.717-1.728)
MLP	1.064 (0.988-1.140)	-0.845 (-1.958-0.267)	0.946 (0.911-0.980)	0.568 (0.062-1.073)

Notes: A perfectly calibrated model would have slope = 1 and intercept = 0. Values > 1 or < 1 indicate over- or under-dispersion of predictions, respectively. 95% confidence intervals are shown in parentheses.

Abbreviations: CI, confidence interval; GAM, Generalized Additive Model; RF, Random Forest; XGBoost, eXtreme Gradient Boosting; LightGBM, Light Gradient Boosting Machine; SVR, Support Vector Regression; KNN, K- Nearest Neighbors; MLP, Multi- Layer Perceptron.

Supplementary Figure 2 Calibration plots for all models (internal validation set)

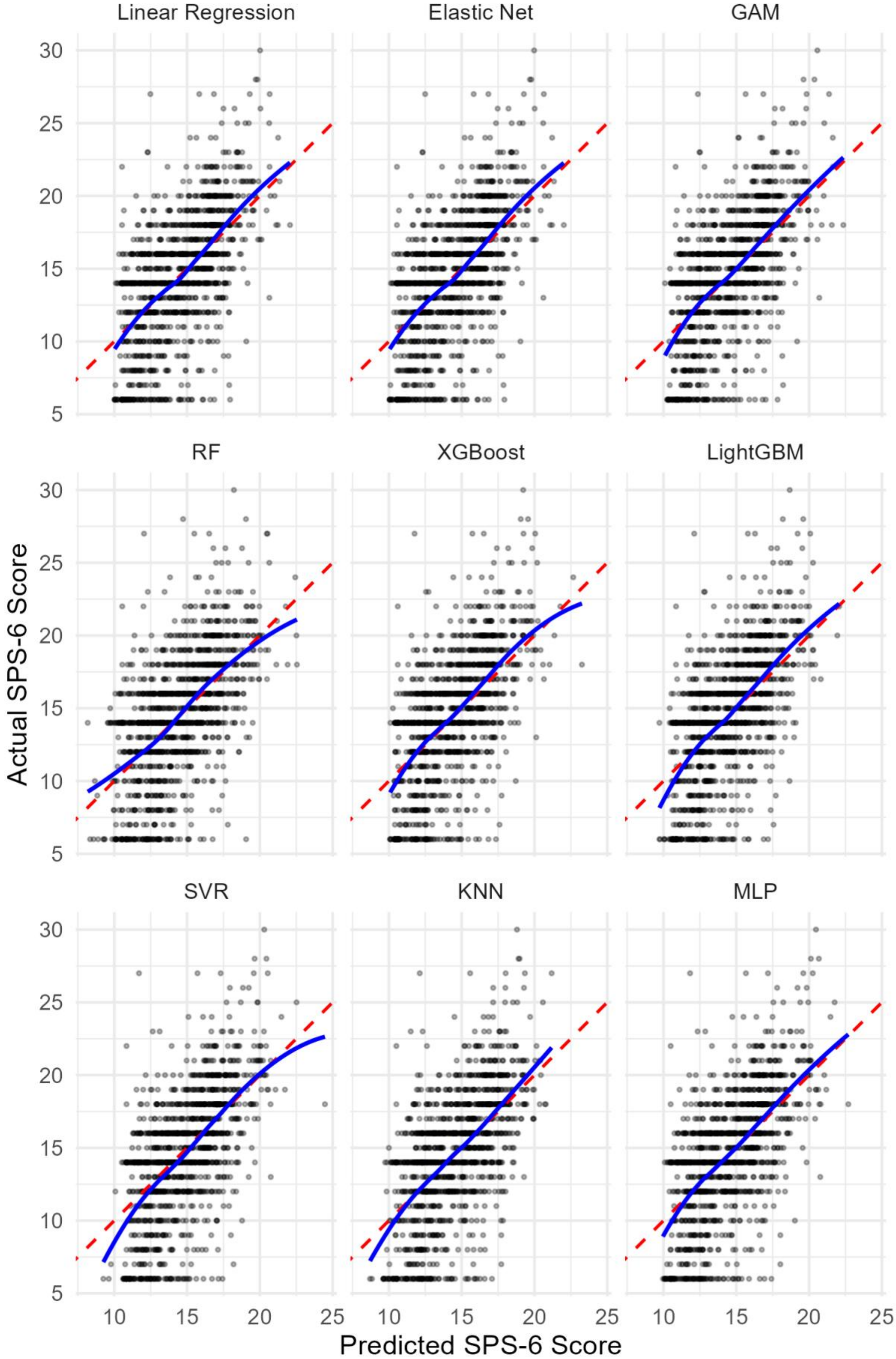

Abbreviations: GAM, Generalized Additive Model; RF, Random Forest; XGBoost, eXtreme Gradient Boosting; LightGBM, Light Gradient Boosting Machine; SVR, Support Vector Regression; KNN, K- Nearest Neighbors; MLP, Multi- Layer Perceptron.

Supplementary Figure 3 Risk stratification violin plots across models (internal validation set)

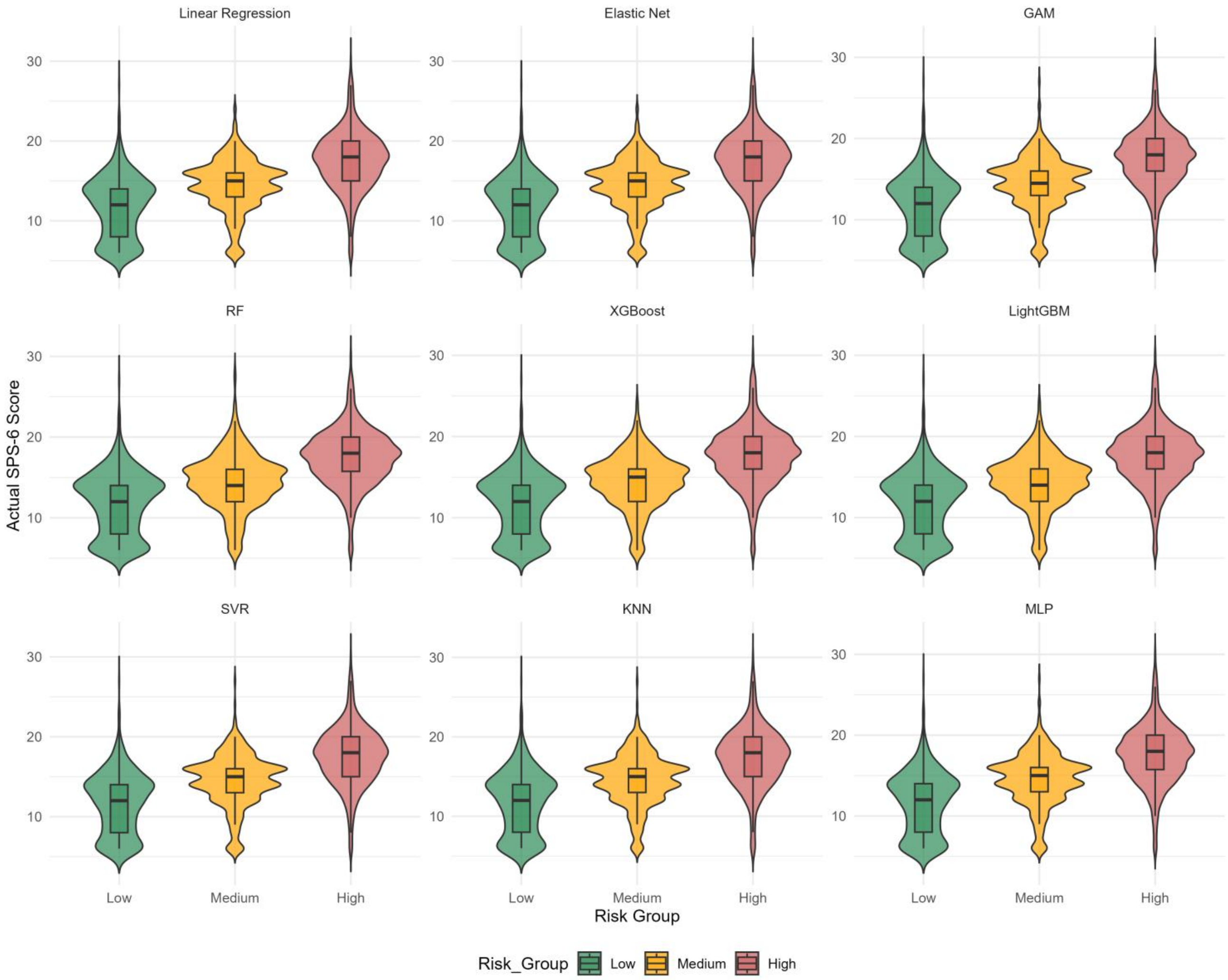

Notes: Risk groups were defined by tertile thresholds of each model's predicted SPS-6 scores. Low = 1st tertile, Medium = 2nd tertile, High = 3rd tertile.

Abbreviations: GAM, Generalized Additive Model; RF, Random Forest; XGBoost, eXtreme Gradient Boosting; LightGBM, Light Gradient Boosting Machine; SVR, Support Vector Regression; KNN, K- Nearest Neighbors; MLP, Multi- Layer Perceptron.

Supplementary Figure 4 Risk stratification scatter plots for all models (internal validation set)

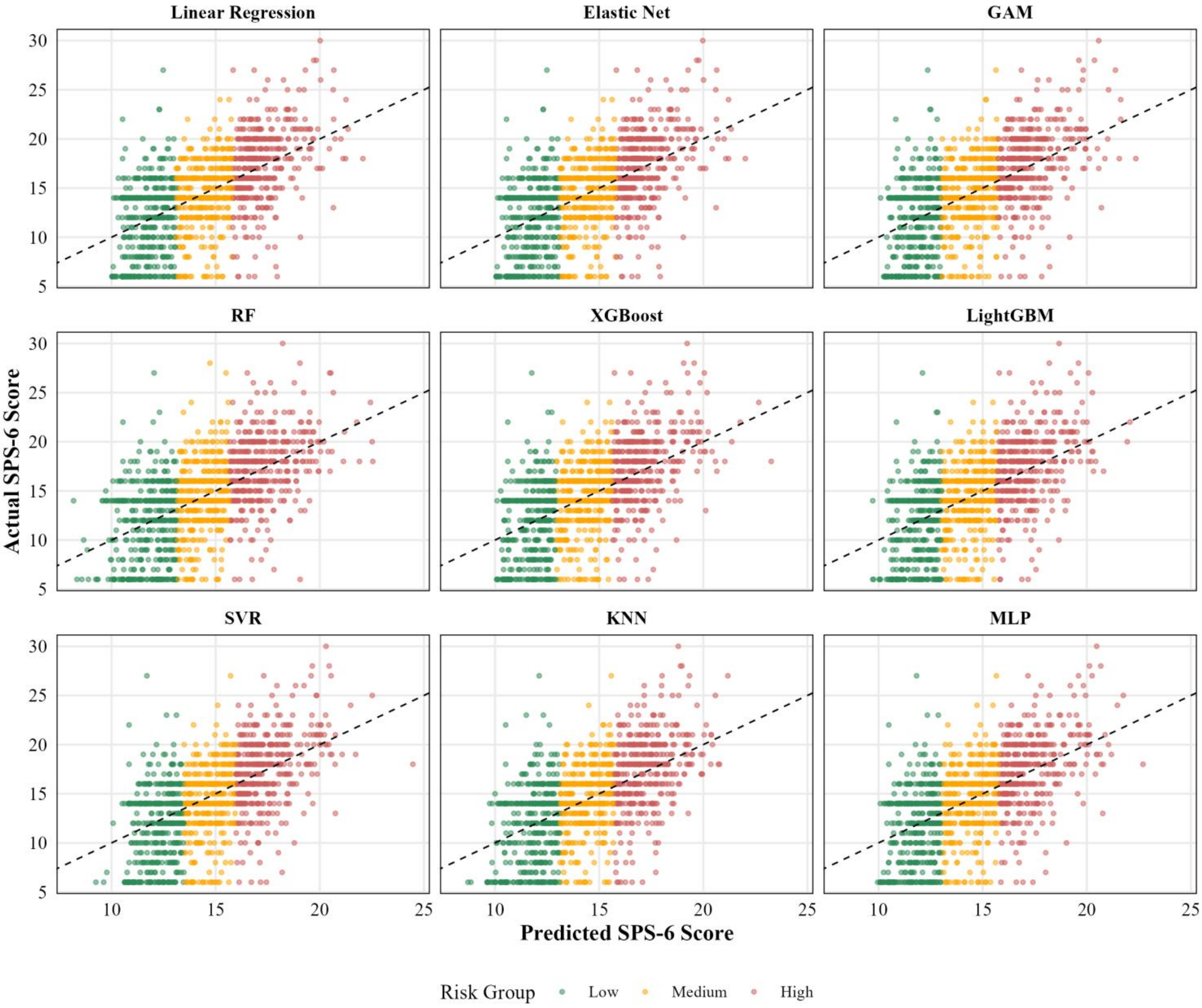

Abbreviations: GAM, Generalized Additive Model; RF, Random Forest; XGBoost, eXtreme Gradient Boosting; LightGBM, Light Gradient Boosting Machine; SVR, Support Vector Regression; KNN, K- Nearest Neighbors; MLP, Multi- Layer Perceptron.

Supplementary Table 7 Cluster-robust standard errors (department-level clustering)

Coefficient	SE (Standard)	SE (Cluster-Robust)	Ratio
Intercept	0.3060	0.2751	0.90
Workplace violence exposure (Yes)	0.1189	0.0701	0.59
Involved in night shifts (Yes)	0.0770	0.1009	1.31
PSSS	0.0031	0.0027	0.88
MBI-GS	0.0356	0.0367	1.03
Organizational Climate	0.0030	0.0021	0.69

Notes: Cluster-robust standard errors estimated using the Huber-White sandwich estimator (HC1) with department as the clustering variable. All coefficients remained statistically significant at $P < 0.001$. Ratio = cluster-robust SE / standard SE. Values < 1 indicate that clustering did not inflate standard errors; the only ratio > 1 (Involved in night shifts, 1.31) was modest and did not affect statistical significance.

Supplementary Table 8 GAM model specification and coefficient estimates

**Panel A. Parametric coefficients**				
**Term**	**Estimate**	**SE**	**t**	**P**
Intercept	15.299	0.114	133.773	< 0.001
Workplace violence exposure (Yes)	−0.644	0.119	−5.415	< 0.001
Involved in night shifts (Yes)	−0.494	0.077	−6.438	< 0.001
**Panel B. Smooth terms**				
**Smooth term**	**EDF**	**Ref.df**	**F**	**P**
s(PSSS)	2.915	3.66	22.045	< 0.001
s(MBI-GS)	3.733	4.658	377.11	< 0.001
s(Organizational Climate)	7.232	8.038	11.213	< 0.001
**Panel C. Model fit**				
**Metric**	**Value**			
Adjusted R^2	0.336			
Deviance explained	33.70%			
Scale estimate	12.241			
n (training set)	9,326			
Method	REML			
Smooth type	Thin-plate regression spline			
Family	Gaussian (identity link)			

Notes: Notes for Panel A: In the GAM, the parametric coefficients for categorical predictors represent the conditional effect when all smooth terms are at their mean (zero). Because the smooth terms s(PSSS), s(MBI-GS), and s(Organizational Climate) are correlated with the categorical predictors, the parametric coefficients for workplace violence exposure and involved in night shifts do not represent the total marginal effects of these variables and may differ in direction and magnitude from the total-effect estimates in the linear regression (Supplementary Table 2). The total effects of all five predictors, as captured by the GAM's combined parametric and smooth components, are visualized through SHAP dependence plots (Figure 2) and partial dependence plots (Figure 3), which consistently show that workplace violence exposure and involvement in night shifts are associated with higher predicted SPS-6 scores.

Supplementary Table 9 Top five SHAP interaction pairs for each risk group

Risk Group	Feature1	Feature2	Interaction Value	Rank
Low	MBI-GS	PSSS	0.992	1
Low	MBI-GS	Organizational Climate	0.874	2
Low	Involved in night shifts	MBI-GS	0.545	3
Low	Organizational Climate	PSSS	0.185	4
Low	MBI-GS	Workplace Violence Exposure	0.168	5
Medium	MBI-GS	PSSS	0.201	1
Medium	MBI-GS	Organizational Climate	0.195	2
Medium	Involved in night shifts	MBI-GS	0.168	3
Medium	MBI-GS	Workplace Violence Exposure	0.117	4
Medium	Organizational Climate	PSSS	0.074	5
High	MBI-GS	Organizational Climate	0.737	1
High	MBI-GS	PSSS	0.616	2
High	Involved in night shifts	MBI-GS	0.399	3
High	MBI-GS	Workplace Violence Exposure	0.369	4
High	Organizational Climate	PSSS	0.122	5

Notes: Interaction Value = mean |SHAP_i × SHAP_j| for each pair, estimated within the respective risk stratum.

Abbreviations: PSSS, Perceived social support scale; MBI-GS, Maslach Burnout Inventory-General Survey.

Supplementary Table 10 Mean absolute SHAP values for five core predictors by risk group

Feature	Mean|SHAP| Low	Mean|SHAP| Medium	Mean|SHAP| High	Mean|SHAP| Avg
MBI-GS	2.211	0.767	1.892	1.623
PSSS	0.443	0.27	0.317	0.343
OC	0.399	0.262	0.357	0.34
INS	0.247	0.223	0.21	0.227
WVE	0.076	0.133	0.191	0.134

Notes: Risk groups were defined by tertiles of GAM-predicted SPS-6 scores. SHAP values were computed on 1500 randomly sampled test-set observations.

Abbreviations: SHAP, SHapley Additive exPlanations; MBI-GS, Maslach Burnout Inventory-General Survey; PSSS, Perceived social support scale; OC, Organizational Climate; INS, Involved in night shifts; WVE, Workplace violence exposure; Avg, average across Low/Medium/High groups.

Supplementary Table 11 Partial dependence values at key percentiles of continuous predictors

Variable	Variable Value	Percentile	Predicted SPS-6 Score
PSSS	12	Min	15.231
PSSS	36.828	5% (38)	15.095
PSSS	49.241	25% (50)	14.957
PSSS	61.655	50% (62)	14.642
PSSS	71.586	75% (72)	14.311
PSSS	84	95% (84)	13.752
MBI-GS	0	Min	11.897
MBI-GS	0.207	5% (0.135)	12.092
MBI-GS	1.034	25% (0.945)	12.995
MBI-GS	2.069	50% (2.02)	14.445
MBI-GS	2.897	75% (3)	15.732
MBI-GS	4.138	95% (4.2)	17.935
MBI-GS	6	Max	21.131
Organizational Climate	17	Min	15.019
Organizational Climate	40.448	Peak	16.116
Organizational Climate	55.103	5% (55)	14.812
Organizational Climate	66.828	25% (68)	15.052
Organizational Climate	84.414	50% (84)	14.557
Organizational Climate	96.138	75% (96)	13.824
Organizational Climate	102	95% (102)	14.192

Notes: Partial dependence values represent the expected predicted SPS-6 score when the focal variable is fixed at the given value, averaged over the joint distribution of all other predictors in the training set.

Abbreviations: SPS-6, Stanford Presenteeism Scale; PSSS, Perceived social support scale; MBI-GS, Maslach Burnout Inventory-General Survey.

Supplementary Table 12 Summary of two-way interaction partial dependence predictions from the GAM model

Interaction Pair	Variables Involved	Min Predicted SPS-6	Max Predicted SPS-6	Range
MBI-GS × Organizational Climate	MBI-GS, Organizational Climate	11.21	22.7	11.49
PSSS × MBI-GS	PSSS, MBI-GS	11.09	21.81	10.71
PSSS × Organizational Climate	PSSS, Organizational Climate	13.07	16.8	3.74

Notes: Predicted SPS-6 values were derived from the final GAM using partial dependence with a grid resolution of 20 × 20. Range = Max − Min.

Abbreviations: GAM, Generalized Additive Model; SPS-6, Stanford Presenteeism Scale; PSSS, Perceived Social Support Scale; MBI-GS, Maslach Burnout Inventory-General Survey.

Supplementary Table 13 Global metrics of risk-stratified networks

Risk Group	Nodes	Total Edges	Nonzero Edges	Density	Positive Edges	Negative Edges
Low	14	91	50	0.549	30	20
Medium	14	91	61	0.670	41	20
High	14	91	68	0.747	37	31

Notes: All networks contained the same 14 nodes; total possible undirected edges = 14 × 13 / 2 = 91.

Supplementary Table 14 Node-level centrality indices by risk group

Node	Strength	Expected Influence	Betweenness	Risk Group
W1	0.032	0.002	0	Low
W2	0.001	-0.001	0	Low
S1	0.57	-0.105	76	Low
S2	0.314	-0.058	0	Low
P1	0.778	0.671	0	Low
P2	0.953	0.885	0	Low
P3	1.114	0.976	44	Low
M1	0.536	0.178	38	Low
M2	0.328	0.167	0	Low
M3	0.398	-0.012	0	Low
O1	0.725	0.691	8	Low
O2	1.034	1.015	18	Low
O3	0.938	0.905	18	Low
O4	0.883	0.735	54	Low
W1	0.361	0.249	0	Medium
W2	0.006	-0.003	0	Medium
S1	0.624	-0.139	24	Medium
S2	0.505	-0.176	0	Medium
P1	0.865	0.762	6	Medium
P2	1.018	0.986	0	Medium
P3	1.126	1.077	22	Medium
M1	1.111	0.089	48	Medium
M2	0.608	0.133	0	Medium
M3	1.383	-0.26	82	Medium
O1	0.737	0.693	0	Medium
O2	1.135	1.084	20	Medium
O3	1.097	1.097	28	Medium
O4	0.981	0.585	0	Medium
W1	0.239	0.033	0	High
W2	0.033	-0.004	0	High
S1	0.876	-0.14	50	High
S2	0.58	-0.226	0	High
P1	0.968	0.704	26	High
P2	0.992	0.943	0	High
P3	1.046	0.83	16	High
M1	1.248	0.534	58	High
M2	1.122	0.34	6	High
M3	1.038	-0.416	10	High
O1	0.823	0.757	0	High
O2	1.175	1.053	42	High
O3	1.094	0.904	4	High

O4	1.002	0.57	32	High

Notes: Strength = sum of absolute edge weights connected to the node. Expected Influence = sum of signed edge weights (positive minus negative). Betweenness = number of times the node lies on the shortest path between any two other nodes. Risk groups were defined by tertiles of GAM-predicted SPS-6 scores.

Abbreviations: W1, Workplace violence exposure; W2, Involved in night shifts; S1, Completing work; S2, Avoiding distractions; P1, Family support; P2, Friend support; P3, Significant other support; M1, Emotional exhaustion; M2, Depersonalization; M3, Reduced personal accomplishment; O1, Hospital management; O2, Department leadership & communication; O3, Work cooperation & prospects; O4, Doctor-patient understanding & communication; PSSS, Perceived social support scale; MBI-GS, Maslach Burnout Inventory-General Survey; SPS-6, Stanford Presenteeism Scale; OC, Organizational Climate.

Supplementary Figure 5 Bootstrapped edge weight 95% CIs for each risk group network

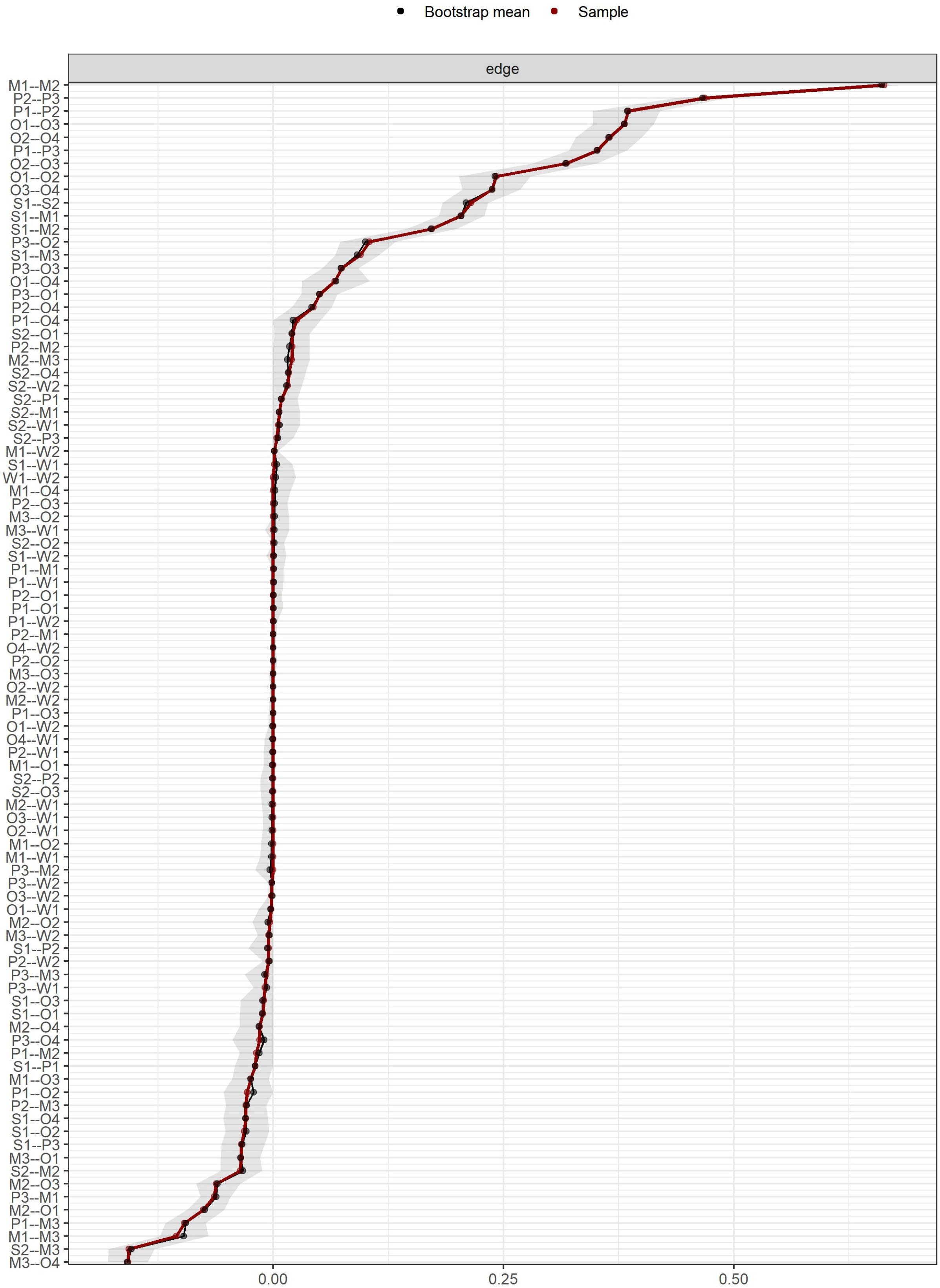

Note: Non-parametric bootstrap (2,000 iterations) 95% confidence intervals for edge weights in the regularized partial correlation networks. Red lines indicate sample estimates; grey areas indicate bootstrapped 95% CIs. Edges are ordered by decreasing absolute weight. Narrow CIs for the strongest edges support estimation precision.

Supplementary Figure 6 Case-dropping bootstrap CS-coefficient plots

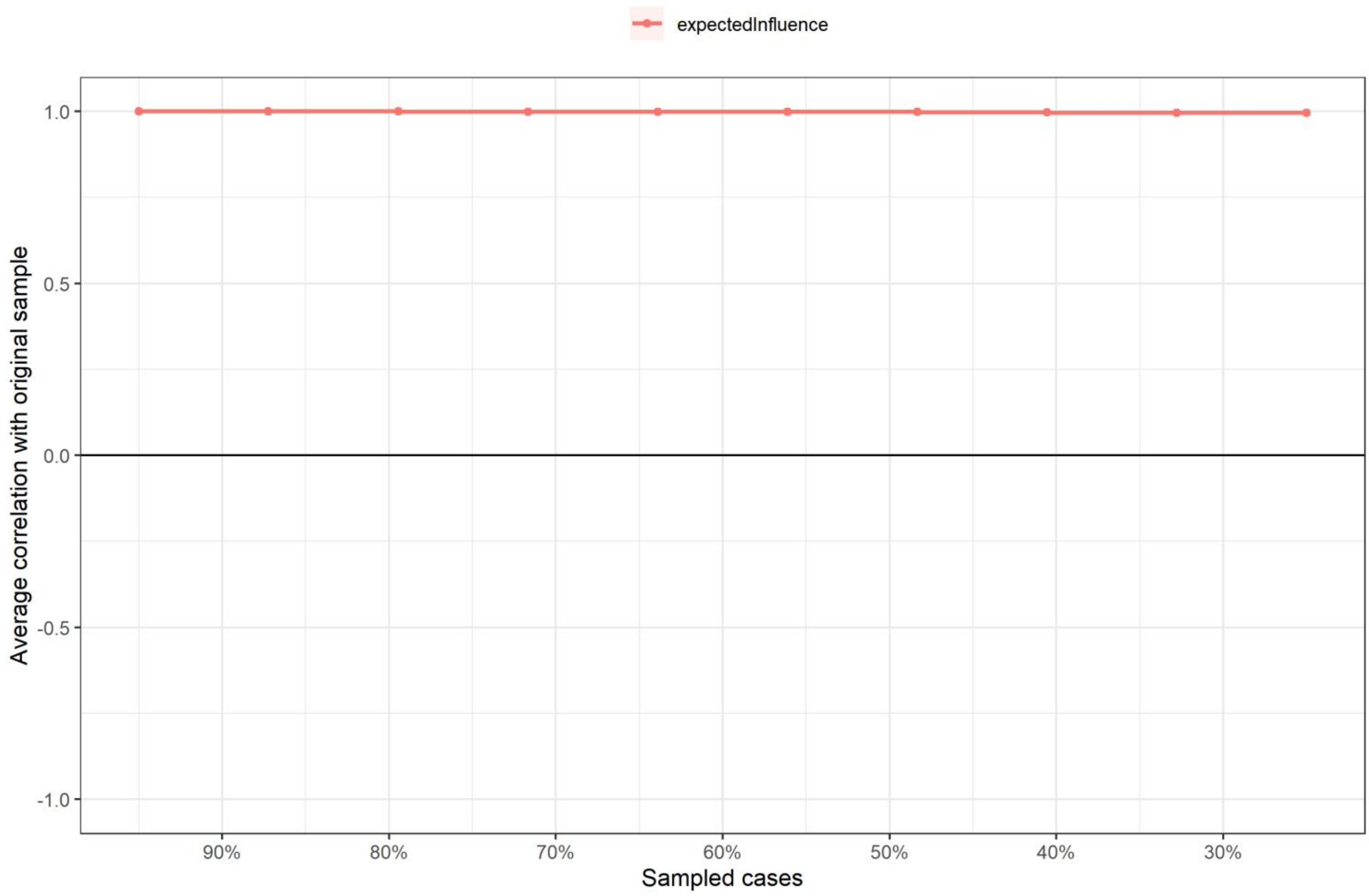

Note: Case-dropping bootstrap (2,000 iterations) stability of strength and expected influence centrality indices. The x-axis shows the proportion of cases dropped; the y-axis shows the correlation between original and subset centralities. CS-coefficients for strength were 0.67 (low-risk), 0.59 (medium-risk), and 0.62 (high-risk); for expected influence they were 0.71, 0.63, and 0.67—all exceeding the recommended 0.50 threshold (and well above the 0.25 minimum), indicating acceptable to excellent stability.

Supplementary Figure 7 Bootstrapped difference tests for edge weights and node strengths

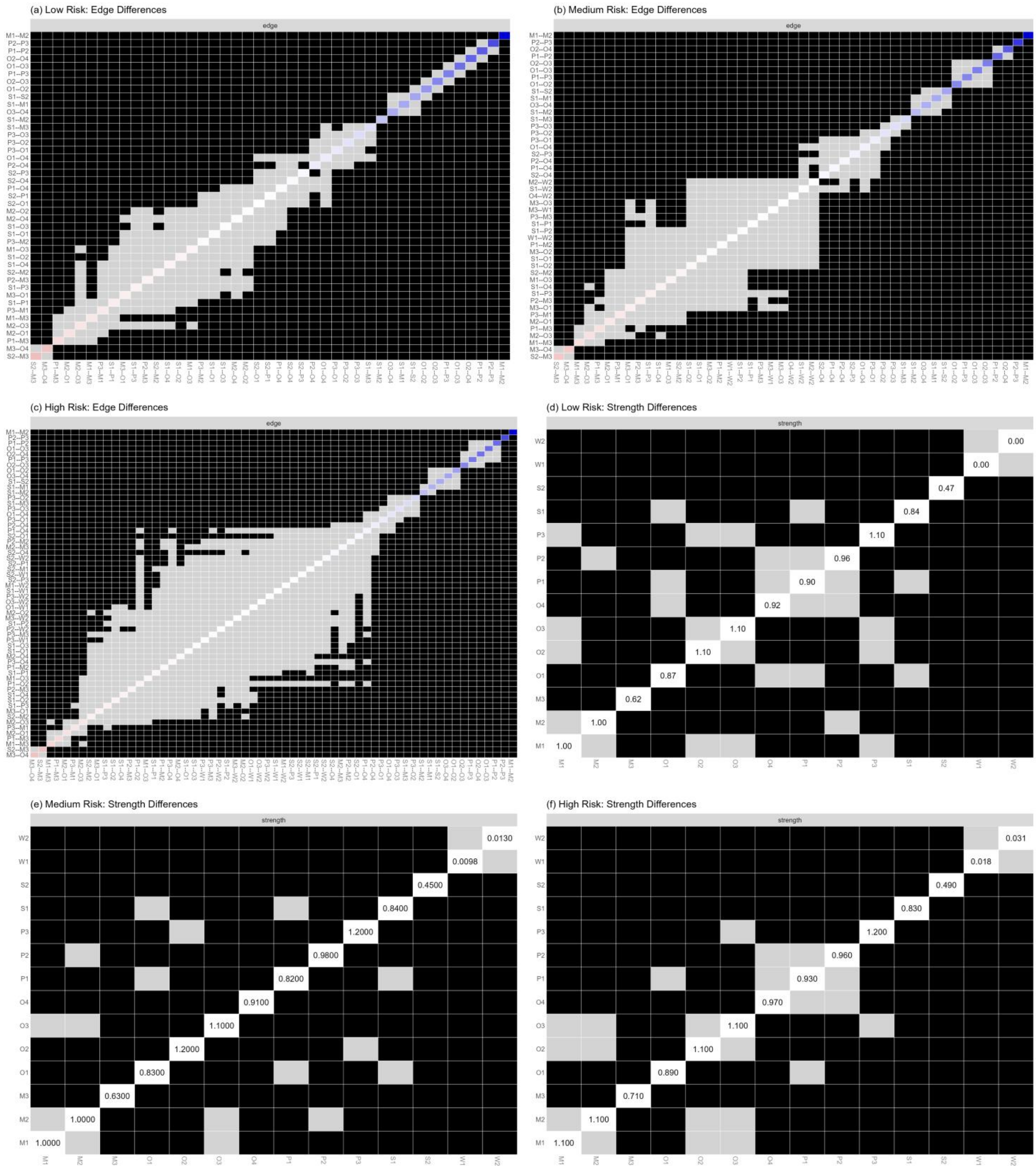

Notes: Bootstrapped difference tests for edge weights (left column) and node strength centrality (right column). Black boxes indicate pairs that differ significantly (α = 0.05); grey boxes indicate non-significant differences. Core network edges and central nodes varied significantly from peripheral ones across all risk groups.

Supplementary Figure 8 Sensitivity networks with EBIC tuning parameters γ = 0.25, 0.50, and 0.75

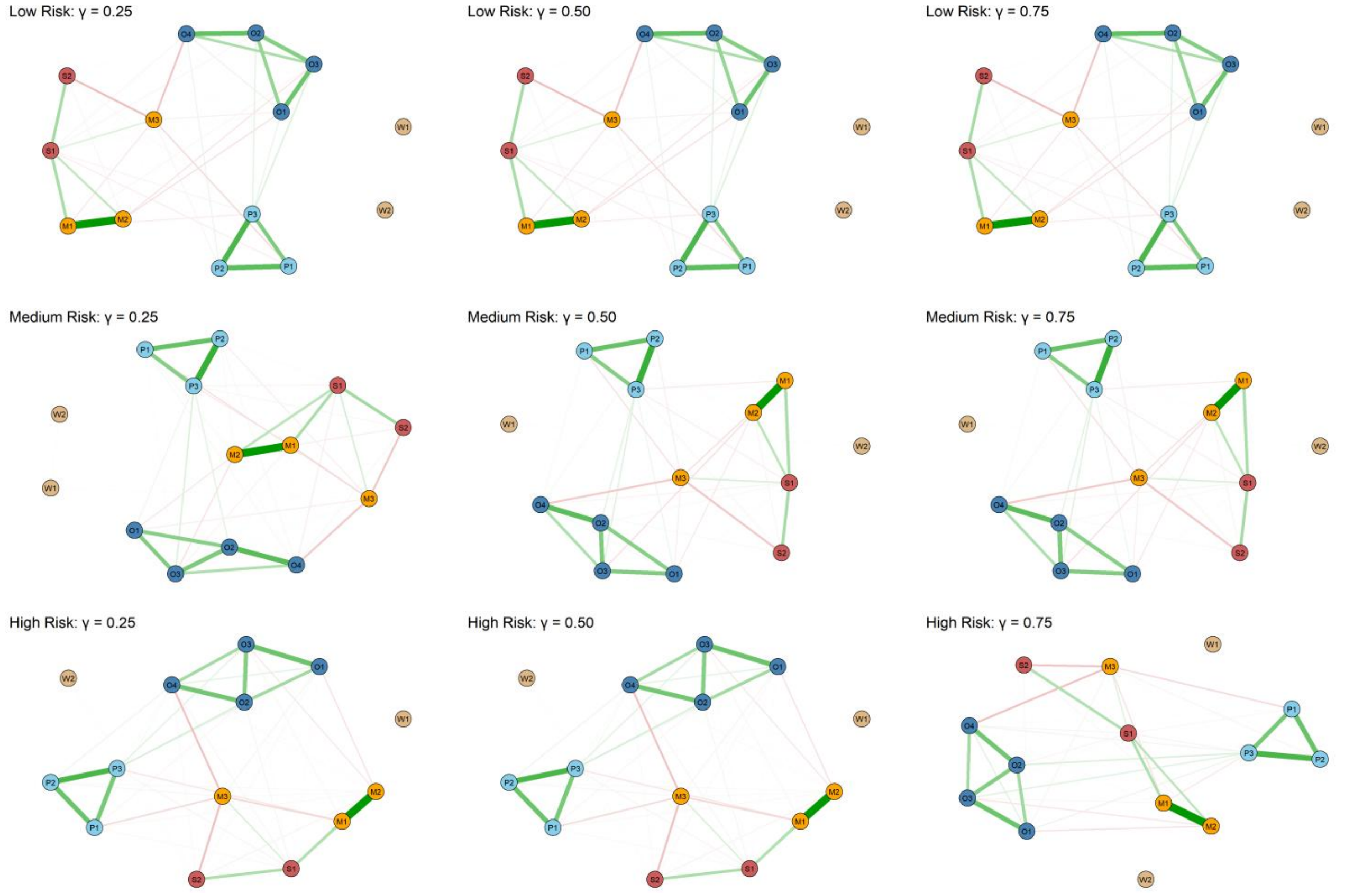

Notes: Regularized partial correlation networks estimated with EBIC tuning parameters γ = 0.25 (sparser), γ = 0.50 (default), and γ = 0.75 (denser). The risk-dependent gradient in network density (Low < Medium < High) was preserved across all γ values, and core node identification remained consistent (see Supplementary Table for densities).

Supplementary Table 15 SHAP main effects by actual SPS-6 risk group

Risk Group	MBI-GS	PSSS	Organizational Climate	Involved in night shifts	Workplace Violence Exposure
Low	1.665	0.381	0.343	0.234	0.108
Medium	1.345	0.283	0.322	0.227	0.114
High	1.726	0.321	0.345	0.216	0.184

Notes: Mean |SHAP| values computed within each risk group defined by tertiles of actual (observed) SPS-6 scores in the internal validation set. The pattern of "high at both ends, low in the middle" for MBI-GS is consistent with the primary analysis using GAM-predicted tertiles.

Supplementary Figure 9 SHAP feature importance: predicted vs. actual SPS-6 tertiles

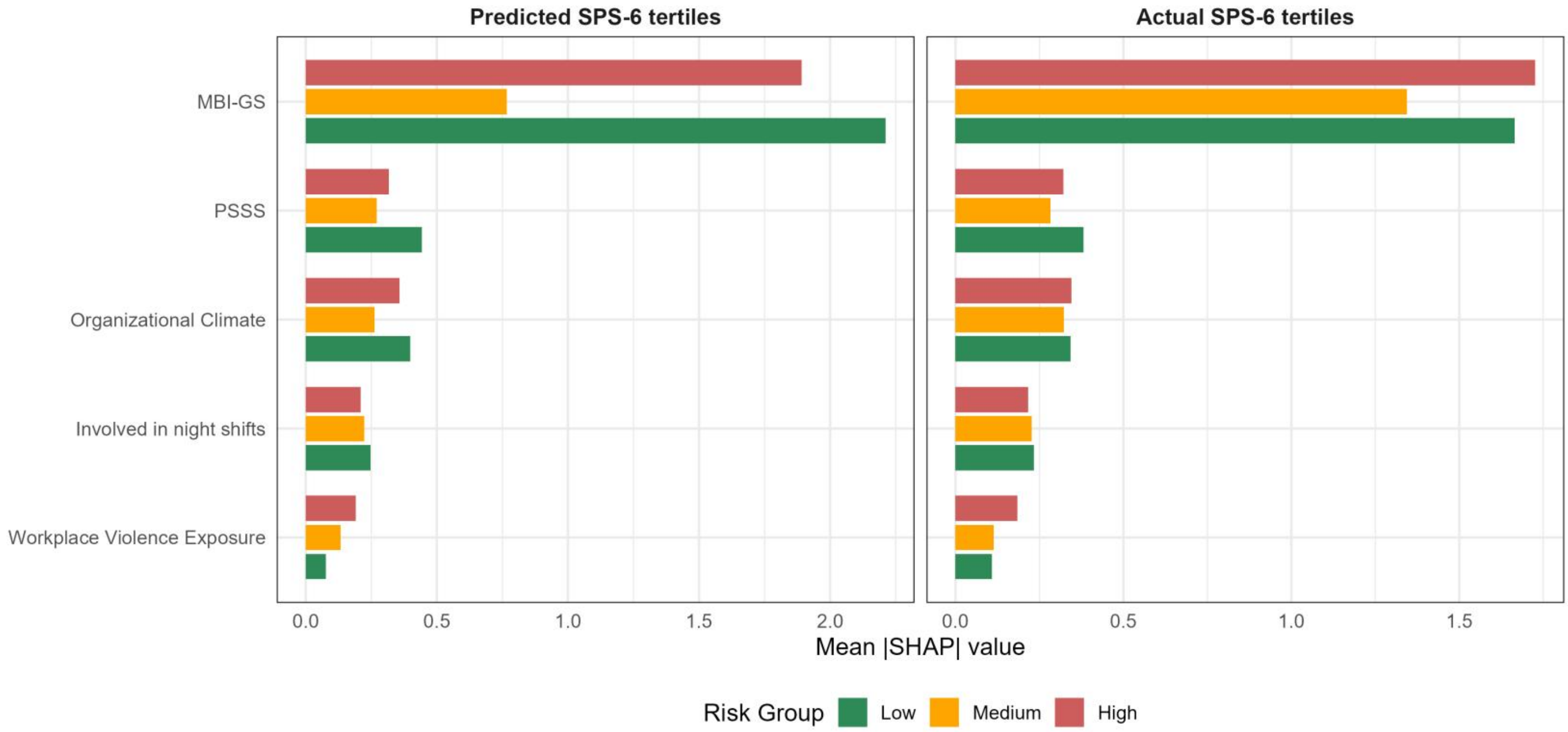

Supplement: Supplementary file 1 [file JONM-2026-6257117-s002.pdf]
