# Supplementary material for: Development and External Validation of a Machine Learning Model for Risk Stratification of Presenteeism in Clinical Nurses: A Multicenter Cross‐Sectional Study
Source: J Nurs Manag. 2026 Sep 9;2026:6257117. doi: 10.1155/jonm/6257117 (PMC13559007; doi:10.1155/jonm/6257117)
Supplement: Supplementary file 2 [file JONM-2026-6257117-s001.doc]

STROBE Statement—Checklist of items that should be included in reports of ***cross-sectional studies***

|  | Item No | Recommendation | Reported on Page |
| --- | --- | --- | --- |
| **Title and abstract** | 1 | (*a*) Indicate the study’s design with a commonly used term in the title or the abstract | Title, p.1 ("A Multicenter Cross‑Sectional Study") |
|  |  | (*b*) Provide in the abstract an informative and balanced summary of what was done and what was found | Abstract, p.2 |
| Introduction | | |  |
| Background/rationale | 2 | Explain the scientific background and rationale for the investigation being reported | Section 1 (Introduction), pp.2–3 |
| Objectives | 3 | State specific objectives, including any prespecified hypotheses | Section 1 (Introduction), final paragraph, p.3 |
| Methods | | |  |
| Study design | 4 | Present key elements of study design early in the paper | Section 2.1, p.4 |
| Setting | 5 | Describe the setting, locations, and relevant dates, including periods of recruitment, exposure, follow-up, and data collection | Section 2.1, p.4 |
| Participants | 6 | (*a*) Give the eligibility criteria, and the sources and methods of selection of participants | Section 2.1, p.4 |
| Variables | 7 | Clearly define all outcomes, exposures, predictors, potential confounders, and effect modifiers. Give diagnostic criteria, if applicable | Section 2.3, pp.4–5 |
| Data sources/ measurement | 8* | For each variable of interest, give sources of data and details of methods of assessment (measurement). Describe comparability of assessment methods if there is more than one group | *Section 2.3, pp.4–5* |
| Bias | 9 | Describe any efforts to address potential sources of bias | Section 2.1 (mandatory response checks, IP restrictions); Section 2.4.3 (cluster-robust SE); Section 2.4.5 (network robustness diagnostics) |
| Study size | 10 | Explain how the study size was arrived at | Section 2.1, p.4 (21,272 distributed → 19,729 valid; sample size determined by convenience sampling across 153 hospitals) |
| Quantitative variables | 11 | Explain how quantitative variables were handled in the analyses. If applicable, describe which groupings were chosen and why | Section 2.4.1 (normality assessment, presentation); Section 2.4.4 (risk tertiles); Section 2.4.6 (actual-SPS-6 tertiles for sensitivity analysis) |
| Statistical methods | 12 | (*a*) Describe all statistical methods, including those used to control for confounding | Section 2.4, pp.5–6 |
|  |  | (*b*) Describe any methods used to examine subgroups and interactions | Section 2.4.4 (SHAP interactions); Section 2.4.5 (network analysis by risk group); Section 2.4.6 (sensitivity analysis) |
|  |  | (*c*) Explain how missing data were addressed | Section 2.4.1, p.5 (electronic questionnaire mandatory response checks → no missing data) |
|  |  | (*d*) If applicable, describe analytical methods taking account of sampling strategy | N/A |
|  |  | (*e*) Describe any sensitivity analyses | Section 2.4.3 (cluster-robust SE); Section 2.4.5 (EBIC γ = 0.25, 0.75); Section 2.4.6 (actual SPS-6 tertiles) |
| Results | | |  |
| Participants | 13* | (a) Report numbers of individuals at each stage of study—eg numbers potentially eligible, examined for eligibility, confirmed eligible, included in the study, completing follow-up, and analysed | Section 3.1, p.7; Table 1 (n = 19,729; derivation: 13,323; external validation: 6,406) |
|  |  | (b) Give reasons for non-participation at each stage | Section 2.1, p.4 (1,543 excluded for abnormal completion time or patterned responses) |
|  |  | (c) Consider use of a flow diagram | Not included |
| Descriptive data | 14* | (a) Give characteristics of study participants (eg demographic, clinical, social) and information on exposures and potential confounders | Table 1, pp.7–8 |
|  |  | (b) Indicate number of participants with missing data for each variable of interest | Section 2.4.1, p.5 (no missing values for any analyzed variable) |
| Outcome data | 15* | Report numbers of outcome events or summary measures | Table 1 (SPS-6 median, Q1, Q3); Section 3.2.2 (risk group cutoffs and means) |
| Main results | 16 | (*a*) Give unadjusted estimates and, if applicable, confounder-adjusted estimates and their precision (eg, 95% confidence interval). Make clear which confounders were adjusted for and why they were included | Supplementary Table 2 (multivariable linear regression coefficients with 95% CIs) |
|  |  | (*b*) Report category boundaries when continuous variables were categorized | Section 3.2.2, p.9 (risk group tertile thresholds: ≤12.99, 12.99–15.71, >15.71) |
|  |  | (*c*) If relevant, consider translating estimates of relative risk into absolute risk for a meaningful time period | N/A (continuous outcome, not applicable) |
| Other analyses | 17 | Report other analyses done—eg analyses of subgroups and interactions, and sensitivity analyses | Section 3.3 (SHAP, PDP); Section 3.4 (risk-stratified network analysis); Section 3.5 (sensitivity analysis with actual SPS-6 tertiles) |
| Discussion | | |  |
| Key results | 18 | Summarise key results with reference to study objectives | Section 4.1, p.15 |
| Limitations | 19 | Discuss limitations of the study, taking into account sources of potential bias or imprecision. Discuss both direction and magnitude of any potential bias | Section 4.5, pp.17–18 |
| Interpretation | 20 | Give a cautious overall interpretation of results considering objectives, limitations, multiplicity of analyses, results from similar studies, and other relevant evidence | Sections 4.2–4.3, pp.15–17 |
| Generalisability | 21 | Discuss the generalisability (external validity) of the study results | Section 4.5, pp.17–18 (single province limitation; independent external validation as strength) |
| Other information | | |  |
| Funding | 22 | Give the source of funding and the role of the funders for the present study and, if applicable, for the original study on which the present article is based | Funding section, p.19 |

*Give information separately for exposed and unexposed groups.

**Note:** An Explanation and Elaboration article discusses each checklist item and gives methodological background and published examples of transparent reporting. The STROBE checklist is best used in conjunction with this article (freely available on the Web sites of PLoS Medicine at http://www.plosmedicine.org/, Annals of Internal Medicine at http://www.annals.org/, and Epidemiology at http://www.epidem.com/). Information on the STROBE Initiative is available at www.strobe-statement.org.
